# Polymeric Hydrogels for In Vitro 3D Ovarian Cancer Modeling

**DOI:** 10.3390/ijms23063265

**Published:** 2022-03-17

**Authors:** Simona Braccini, Chiara Tacchini, Federica Chiellini, Dario Puppi

**Affiliations:** BioLab Research Group, Department of Chemistry and Industrial Chemistry, University of Pisa, UdR INSTM-Pisa, Via Moruzzi 13, 56124 Pisa, Italy; simona.braccini@phd.unipi.it (S.B.); chiara.tacchini97@gmail.com (C.T.)

**Keywords:** 3D cell culture, ovarian cancer, scaffold, polymer, hydrogel

## Abstract

Ovarian cancer (OC) grows and interacts constantly with a complex microenvironment, in which immune cells, fibroblasts, blood vessels, signal molecules and the extracellular matrix (ECM) coexist. This heterogeneous environment provides structural and biochemical support to the surrounding cells and undergoes constant and dynamic remodeling that actively promotes tumor initiation, progression, and metastasis. Despite the fact that traditional 2D cell culture systems have led to relevant medical advances in cancer research, 3D cell culture models could open new possibilities for the development of an in vitro tumor microenvironment more closely reproducing that observed in vivo. The implementation of materials science and technology into cancer research has enabled significant progress in the study of cancer progression and drug screening, through the development of polymeric scaffold-based 3D models closely recapitulating the physiopathological features of native tumor tissue. This article provides an overview of state-of-the-art in vitro tumor models with a particular focus on 3D OC cell culture in pre-clinical studies. The most representative OC models described in the literature are presented with a focus on hydrogel-based scaffolds, which guarantee soft tissue-like physical properties as well as a suitable 3D microenvironment for cell growth. Hydrogel-forming polymers of either natural or synthetic origin investigated in this context are described by highlighting their source of extraction, physical-chemical properties, and application for 3D ovarian cancer cell culture.

## 1. Introduction

Ovarian cancer (OC) is the seventh most common cancer in women [1]. In 2021, there were approximately 21,410 new cases of OC, and 13,770 people died from this disease in the U.S. [2]. Even though the incidence of new cases and mortality rate have declined over the past two decades, OC continues to be the most lethal gynecological cancer in the world. The high death-to-incidence ratio is caused by several factors, including: (i) the advanced stage of the disease at the time of diagnosis because of the asymptomatic nature of OC and the absence of effective screening strategies; (ii) resistance to treatment; (iii) the development of recurrence [3]. The stage of cancer at diagnosis determines the treatment options, with a strong influence on the duration of survival. The sooner OC is detected, the better a patient’s chances of surviving five years after diagnosis. In the case of OC, only 15.7% of patients are diagnosed in the early stage and the 5-year relative survival rate for localized tumor is 92.6%, while in the advanced stage it is 48.6% [2].

Although in recent years there has been a significant increase in research towards new therapeutic targets and strategies, as well as the development of new drugs, the standard treatment of OC since the 1980s includes surgery, with a goal of complete tumor resection, and chemotherapy based on platinum compounds and taxanes [4]. New therapeutic approaches are indeed required to improve OC growth and progression response to treatment. The development of a new drug involves critical and systematic steps that present many challenging aspects. It is estimated that 12–15 years of investigation and huge financial costs are needed to develop a new cancer drug. Unfortunately, the percentage of drugs that receive Food and Drug Administration (FDA) approval is very low (<5%) compared to the number of compounds that enter clinical trials [5,6]. One of the main reasons for drug discovery failure is inappropriate pre-clinical testing methods and in vitro tissue models, due to poor efficacy and safety issues. Traditionally, in vitro drug screening is performed on conventional two-dimensional (2D) systems that do not accurately reproduce the in vivo microenvironment. On the other end, new pre-clinical models based on three-dimensional (3D) cell culture offer in vitro biological microenvironments more closely reproducing those observed in vivo [7]. Cancer tissues are not simply a mass of cells with genetic mutations and uncontrolled growth, but rather a physiologically functioning anatomical unit consisting of a heterogeneous set of cells, blood vessels and their surrounding stroma, referred to as tumor microenvironment [8].

**Figure 1:**
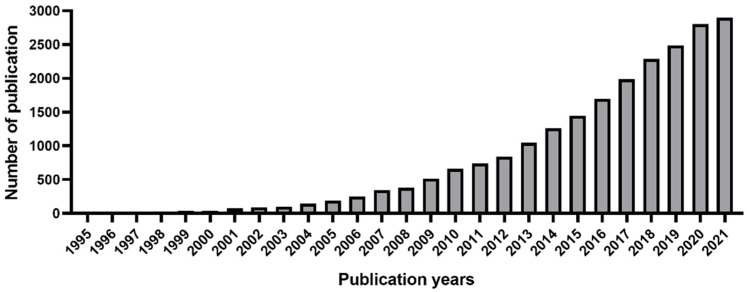
Total number of publications per year with “3D culture” topic. Source: Web of Science: Science Citation Index Expanded [13].

Despite the fact that 3D cell culture models show more realistic morphology and stiffness of the tumoral mass, cell–cell/extracellular matrix (ECM) interactions, and cell sensitivity to drugs and nutrients, they have not yet been universally integrated into the drug development arena. Traditional 2D cell cultures have, therefore, always been predominant in cellular assays used for drugs screening because of their simplicity, reproducibility, and low cost [9,10]. Interest in the development of increasingly convenient and reproducible 3D culture techniques for high-performance phenotypic and pharmacological evaluation has grown significantly only in the last decade (Figure 1) [11].

Hydrogels are widely used in 3D tissue modeling as a promising and versatile class of scaffolding materials thanks to their ability to absorb a large volume of cell culture medium, thus mimicking the highly hydrated environment of the body’s soft organs and tissues [12]. A growing body of literature has been published in the past few years on polymeric hydrogels for OC modeling, by employing different cell lines and macromolecular compounds from either natural sources or synthetic routes. However, to the best of the authors’ knowledge, the literature lacks a paper reviewing this topic. For this reason, this article provides an overview of polymeric hydrogels that have been investigated as scaffolds for 3D cell culture modeling of OC.

## 2. Ovarian Tumor Microenvironment

**Figure 2:**
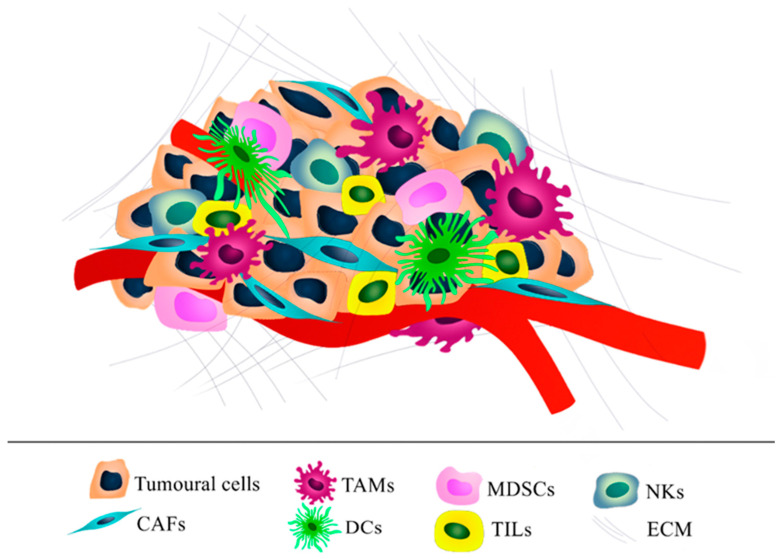
Graphical representation of ovarian tumor microenvironment (TME). In particular, the following components are represented: tumoral cells, cancer-associated fibroblasts (CAFs), tumor-associated macrophages (TAMs), dendritic cells (DCs), myeloid-derived suppressor cells (MDSCs), tumor-infiltrating lymphocytes (TILs), natural killers (NKs) and ECM structural elements.

The ovarian tumor microenvironment (TME) is constituted from non-cellular components, comprising the ECM that provides structural support, and secreted molecules such as chemokines, inflammatory cytokines, matrix metalloproteinases (MMPs), ECM-sequestered growth factors, as well as stromal cells, consisting of cancer cells, cancer-associated fibroblasts (CAFs), endothelial cells (ECs) and immune cells [10]. The dynamic changes among all these components regulate cell adhesion, proliferation, differentiation, migration, survival, as well as disease progression and therapeutic outcome (Figure 2) [5].

The ECM is crucial for maintaining normal function and homeostasis in all tissues and it is composed of fibrous structural proteins, such as collagen, elastin, and fibrillin, glycoproteins (e.g., fibronectin), and proteoglycans (e.g., glycosaminoglycan). In tumorigenesis, an increased deposition of structural proteins occurs, leading to changes in tissue density, and to the formation of a physical barrier that limits mass transport of compounds [10]. In particular, the increasing ECM collagen secretion in epithelial OC, induced by malignant cells, directly correlates with invasiveness and enhanced tumor progression [14]. Moreover, fibronectin produced by fibroblasts and mesothelial cells within TME plays a significant role in the pathogenic process, promoting early metastasis by engaging α5β1 integrins on cancer cells [15], migration and invasion, upregulating the FAK/PI3K/Akt pathway [16], angiogenesis and inhibiting apoptosis [17]. The metastatic process is also enhanced by MMP-2 that cleavages fibronectin and vitronectin into small fragments facilitating cell adhesion to the peritoneal surface [18], as well as by hyaluronan bound from CD44, the major surface receptor of epithelial OC cells [19]. The ECM crosstalk with cells is mainly mediated by integrins, in particular α3β1, αvβ1 and α6β1, proven to be implicated in proliferation, adhesion, migration and invasion of OC cells [16]. For example, integrins can trigger a signaling pathway through the focal adhesion kinase (FAK) that sequesters pro-apoptotic proteins, such as p53 from the apoptotic signaling, determining an increased proliferation and survival. At the same time, other pro-apoptotic molecules (e.g., Bax) are blocked by overexpression of FAK, and several antiapoptotic genes (e.g., Bcl-2) are induced [20]. Nevertheless, also, other cell surface receptors are implied, such as discoidin receptors and syndecans (e.g., syndecan-1, SDC-1), a known promoter of epithelial OC cell proliferation [21].

Cellular behavior is not only determined by the chemical composition of the stroma, but also by its stiffness. During the pathogenetic process, the ECM is altered via desmoplastic response that increases its stiffness through remodeling, enhancing tumor progression by activating a plethora of mechanotransduction pathways. For example, matrix rigidity promotes nuclear translocation of YAP1, an oncogenic transcription factor associated with aggressive metastatic epithelial OC and the disaggregation of multicellular spheroids through a mechanotransduction pathway involving ROCK, actomyosin contractility, and FAK [21]. On the other hand, after the detachment from the primary tumor and the dissemination through the intraperitoneal fluids, OC spheroids have a peculiar tropism mediated from the Rho/ROCK signaling pathway towards soft tissues, such as the adipocyte-rich omentum [22]. Here, a bidirectional interaction between omental adipocytes and cancer cells takes place, causing de-differentiation and reprogramming of adipocytes into cancer-associated adipocytes (CAAs). In this process, cancer cells secrete cytokines and chemokines that induce lipolysis in adipocytes. Then, the released fatty acids are promptly uptaken by cancer cells, which upregulate fatty acid-binding protein 4 (FABP4) in omental metastases, to generate energy by β-oxidation and meet the increasing demand of the rapid tumor growth [23]. At the same time, adipocytes de-differentiate into pre-adipocyte fibroblastoids that secrete adipokines, such as TNF-α, which increases CD44 expression in epithelial OC cells by activating c-Jun N-terminal kinase (JNK) pathway [24], and leptin that promotes the production of MMP-7 by ERK1/2 and JNK1/2 activation [25], enhancing OC cell invasion and metastasis [26].

This metastatic process towards the omentum and peritoneal cavity is sustained by a plethora of different cell types within the TME that contribute creating a permissive environment for tumor proliferation, progression, and immune evasion [27]. In this process, cancer cells recruit and transform the stromal cells that in turn remodel the ECM of the stroma, co-evolving with time in complex and dynamic interactions that assist the metastatic transition of cancer cells (Figure 2).

One of the main cellular components of TME is cancer-associated fibroblasts (CAFs). They usually differentiate from mesenchymal-derived cells but can also transdifferentiate from pericytes or epithelial cells when exposed to platelet-derived growth factor (PDGF), tumor-derived transforming growth factor-β1 (TGF-β1), basic fibroblast growth factor (bFGF), and interleukin-6 (IL-6). CAFs have a peculiar reactive phenotype characterized by the constitutive expression of α-smooth muscle actin (α-SMA), normally expressed by fibroblasts involved in wound healing, where it provides contractile strength, and fibroblast activation protein (FAP), as well as by not undergoing apoptosis or losing the activated phenotype [28]. CAFs enhance tumor progression, invasion and migration via various mechanisms, such as expressing CXCL14, an important factor in promoting cancer growth [29], and increasing the infiltration of FOXP3+ regulatory T lymphocytes (Tregs) in the tumor site, which exerts immune suppression effect [30] and whose presence directly correlates with poor OC survival [31]. CAFs also contribute to vascular stabilization in ovarian and other cancers and remodeling of ECM [32].

The most abundant immune cell population of tumoral stroma are macrophages, named tumor-associated macrophages (TAMs) when present in the TME [31]. TAMs derive from resident macrophages or bone marrow monocytes circulating in the blood and are recruited by the chemokines CCL2, CCL5, and CXCL1 in the TME [33], where they are polarized into the M2 phenotype thanks to TGF-b, IL-10 and IL-4 [34]. In epithelial OC, M2 macrophages are associated with poor prognosis and poor survival, especially because they increase tumor invasion and metastasis formation by producing MMPs, serine proteases and cathepsins that remodel the ECM, enabling cells migration, angiogenesis, and early recurrence [35]. Hence, the maintenance of this phenotype is supported by tumoral cells, sustaining and promoting their survival by producing macrophage colony-stimulating factor (M-CSF) and vascular endothelial growth factor (VEGF) [36]. TAMs also act as suppressors of anti-tumor immune responses by two main strategies. The first is to produce chemokines with an immunosuppressive function, or that recruit only immune cell populations lacking in cytotoxic activity, such as CCL22, also produced by OC cells, that attracts Treg [37]; the second is by expressing on their surface the ligand receptors for cytotoxic T-lymphocyte antigen-4 (CTLA-4) and programmed cell death protein ligand-1 (PD-1), whose activation inhibits cytotoxic function and regulates T-cells’ cycle [38]. Additionally, they stimulate cancer cell growth both directly, producing EGF, IL-6 and tumor necrosis factor (TNF), and indirectly, secreting pro-angiogenic factors [39].

Angiogenesis is the process of new vessel formation, the principal source of nutrient supply and a way out for cancer cell dissemination. The main inducer is VEGF, expressed by tumoral cells in case of oxygen depletion via hypoxia-inducible transcription factors 1a and 2a, and also by TAMs, tumor-associated neutrophils (TANs), and natural killer (NK) cells to sustain tumor vascularization and the metastatic process [31]. Beside angiogenic properties, VEGF can also induce the expression of FasL ligand, a known regulator of T cell apoptosis, on human tumor endothelial cells, leading to the apoptosis of tumor-infiltrating CD8^+^ T cells [40]. VEGF is, therefore, related to malignant ovarian epithelial metastases and poor clinical outcomes [41].

Myeloid-derived suppressor cells (MDSCs) are a heterogeneous group that include myeloid progenitor cells, immature macrophages, granulocytes and dendritic cells also present in the TME. These cells are characterized by the upregulation of both arginase 1 and inducible nitric oxide synthase, resulting in increased production of immunosuppressive nitric oxide and reactive oxygen species, and leading to the suppression of the tumor-directed immune response [42]. Furthermore, these cells also interact with NK infiltrated in the tumor by secreting TGF-β, a master switcher of immune suppression in NK cells [43], implicated in their induction of a pro-angiogenic phenotype [44]. NKs are also directly inhibited by OC cells overexpressing on their surface a high molecular weight mucin, MUC16 (CA125), that prevents the formation of immune synapses between NK cells and OC targets, hence blocking the immune recognition [45].

Another group of cells found in the TME is tumor-infiltrating lymphocytes (TILs), comprising T-cells and Tregs. The primary function of T-cells would be to inhibit cancer development; however, their action is suppressed by Tregs, MDSCs, and TAMs by secreting a plethora of soluble inhibitory factors, such as IL-6, IL-10, and TGF-β, and through the upregulation of inhibitory receptors on cancer cells, enabling the immune-escape [46].

## 3. In Vitro 2D Tumor Cell Culture

**Figure 3:**
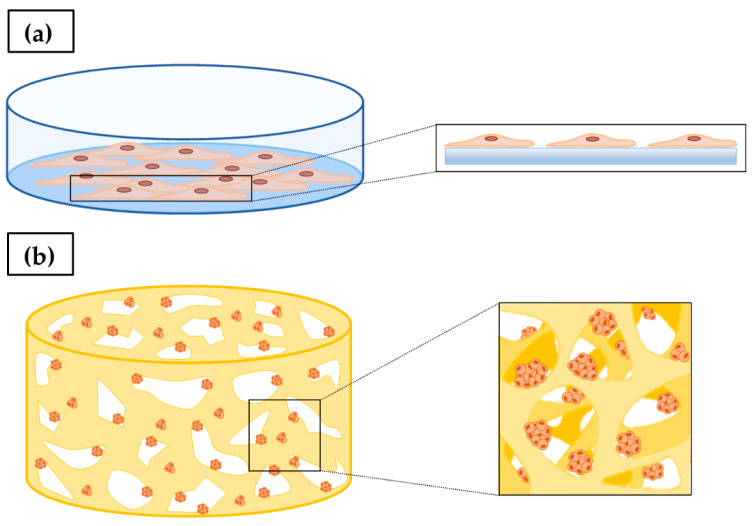
(**a**) Two-dimensional cell culture: cells cultured in a petri dish arranged to form a cellular monolayer with a flat cell shape that does not closely recapitulate the real physiological cell morphology. (**b**) 3D cell culture: in the 3D microenvironment, cells form multicellular aggregates, presenting a morphology and behavior more representative of in vivo systems.

Since the first experiments carried out by Harrison in 1907 during his research on nerve fibers development, 2D cell culture has been continuously developed [47,48]. The advantages of 2D cell culture are mainly the simplicity, cost-effectiveness, and high cell viability [49]. It is routinely employed to study cell biology and physiology, tissue morphology, drug action, disease process and development, and for this reason it is often used in genetic manipulation and biotechnological studies [50,51]. Even if it has allowed for understanding basic aspects of cell tumor biology, monolayer cell culture has numerous disadvantages, mainly related to the poor faithful and realistic representation of the human TME, which as previously highlighted plays a critical role in tumor formation, propagation rate, quiescence and establishment of metastases [52,53,54]. TME can also model tissue therapeutic response and the rise of drug resistance, justifying the recent interest in its components as possible targets for new antitumoral drugs [55,56]. Culturing cells as a monolayer in multi-well plates, tissue culture flasks, or flat petri dishes attached to a polystyrene tissue culture surface does not adequately allow for mimicking the tumor mass natural structure and microenvironment (Figure 3). As a result, 2D cell culture systems typically show altered gene expression and activation of cell signaling pathways, if compared to tumoral tissues in vivo [49,52].

The aforementioned disadvantages highlight an urgent need of in vitro tissue models more closely representing in vivo TME, such as in the case of 3D cell culture systems.

Even if 2D cell culture investigations on OC are still preferred for high-throughput drug screening and tumor biology investigation, increasing attention has been given to the importance of using more accurate pre-clinical models that reduce the gap between in vitro and in vivo TME. Several studies performed on a broad spectrum of OC cell lines have confirmed significant differences in cell morphology, resistance to chemotherapeutic agents and secretion of proliferation markers in cells cultured in 3D versus 2D systems [11,57,58]. Two-dimensional OC cell cultures showed epithelial and fibroblast-like phenotypes. On the other hand, studies carried out on OC spheroids obtained on an ultra-low attachment plate revealed three different cell patterns that could be classified as loose aggregate conformation (A2780, A2780cis, OVCAR3, OAW28, PEA1, PEA2, PEO23 and TO14), compact aggregate structure (PEO1, PEO4, PEO6 and PEO14), and tight spheroid structure (PEO16, OV56, SKOV3 and 59M), based on size, light permeability, and the amount of incorporated cells. Furthermore, OC monolayer and spheroids treated with increasing concentrations of cisplatin, carboplatin, and paclitaxel typically show differences in dose-dependent cell viability reduction. Indeed, most of the OC cell lines cultured using 3D techniques showed greater resistance to these compounds. Moreover, in the case of many OC cell lines, the transition from a 2D to a 3D microenvironment generated changes in the expression of different tumor biomarkers. Brodeur et al. [59] investigated the carboplatin response of six OC cell lines as 2D monolayers, 3D spheroids, or mouse xenograft models. The results obtained from the in vivo study were correlated to the response obtained from the 3D spheroids in four cell lines out of the six tested, while they were correlated to the response obtained from the cell monolayer with only three cell lines. This study clearly demonstrated heterogeneity in therapeutic response when a cell line is grown in different conditions, underlining the need to select an optimized in vitro preclinical model for drug screening studies. This may enhance the exclusion of ineffective drugs in the first phase of preclinical studies, helping to reduce the rate of failed studies, and consequently the cost, time and use of animals in research.

## 4. In Vitro 3D Tumor Cell Culture

Biomimetic 3D multicellular tumor models’ goal is to narrow the gap between 2D in vitro cell culturing and animal testing models. Indeed, 3D systems more accurately mimic the complex cell–cell and cell–ECM interactions, allowing for a better preservation of cellular morphology and heterogeneity characterizing physiological tissues. This is a fundamental requirement since both morphology and cell-environment crosstalk strongly influence gene expression and, therefore, cell behavior and intercellular signaling networks [49,60,61].

Peculiar features and relevant pros and cons of 2D and 3D culture systems are outlined in Table 1.

As discussed below, several in vitro OC studies have been conducted under 3D culture conditions, conventionally divided into two main categories: scaffold-free and scaffold-based models.

### 4.1. Scaffold-Free 3D Cell Culture

Scaffold-free techniques lead to the formation of self-assembled cell units, named spheroids, that remain in suspension in the culture medium without adhering to any surface. During the spheroid formation process, cells secrete their own ECM components through continuous deposition of proteins [54,72]. Spheroids show complex cell-to-cell adhesion and cell-to-matrix interaction, which result in gradients of nutrients, gases, growth factors and signal factors. This kind of structure recapitulates the TME found in real tissues [72,73]. The generation of spheroids is the fastest and most common way to obtain 3D cell models, as evidenced by various techniques described for their formation in literature, as presented below.

**Ultra-low attachment** (**ULA**) techniques are based on the use of low-adhesion plates that are designed with a coating made of a hydrophilic or hydrophobic polymer (e.g., agar, agarose, Matrigel, or poly-2-hydroxyethyl methacrylate) and endowed with a well-defined geometry (e.g., round or V-shaped bottom) to promote the self-aggregation of cells into a single spheroid per well [5,74]. Spheroids assemble starting from 3 days of culture, and are characterized by cells with greater aggressiveness in terms of growth, migration, invasion and in vitro chemotherapy resistance than monolayer cultures [75,76]. For this reason, ULA has been widely used for high-throughput drug screening assays, recently also in the field of OC modeling to find innovative chemotherapy strategies. For instance, promising results were achieved in studies concerning the viral oncolytic properties of three different viruses (i.e., Myxoma, double-deleted vaccinia and Maraba virus), demonstrating that Maraba virus infects, replicates and kills OC cells effectively in cells adhering to a 2D substrate, and to a slightly lesser extent in tumor spheroids [77]. ULA approach was also employed to assess the effect miR-328 and Nectin-4 peptide 10 expression inhibition on the restriction of OC growth and prevention of spheroid formation [78,79]. In addition, a recent study by Hedemann et al. [80] in this context showed increased cytotoxicity of cisplatin when combined with the ADAM17 metalloprotease inhibitor GW280264X.

**The hanging drop** (**HD**) technique principle has been widely employed since 1994 for the formation of multicellular spheroids. Initially, the hanging drop technique was performed by dropping a small volume of cell suspension onto the lid of a Petri dish. Subsequently, the lid was quickly flipped over on the plate containing culture medium or PBS to maintain a moist atmosphere. This allowed for the formation of drops on the lid containing cells, which did not fall due to surface tension. Under the action of the gravity, the cells were forced to accumulate at the apex of the drop, aggregate and proliferate to form spheroids. In recent years, special hanging drop plates (HDPs) have been designed to replace Petri dishes, allowing for the production of multiple 3D spheroids per plate [7,71]. High viability multicellular OC spheroids were obtained by means of novel 384 wells HDP, starting from a limited number of seeded cells (10–100 cells/well) [81]. For this reason, the HDP technique has been selected for the formation of spheroids used in the screening of already known anticancer agents (e.g., cisplatin and placlitaxel [81,82]) and innovative therapeutic strategies, e.g., niraparib and olaparib, two poly(ADP-ribose) polymerase inhibitors [83]. In both cases, a significantly lower cytotoxic activity was observed in comparison to cell monolayer conditions. Indeed, the close relationship between OC ability to form spheroids in vivo and its resistance to chemotherapy is well known. Spheroids obtained through this technique are also used to investigate the invasiveness and migration of OC cells. A study by Sodek et al. [84] verified the close link between the 3D tumor cell conformation and its invasive capacity, highlighting the need of new therapeutic strategies to prevent the formation of spheroids.

**Agitation-based approaches** are also widely employed to culture cell spheroids. Cells are kept in suspension by agitation under the action of a mechanical stirrer or through the direct bioreactor rotation (e.g., wall-rotating system). Continuous agitation is essential to prevent cell adhesion to the walls/bottom of the bioreactor and promote cell–cell interactions. The main devices used for cell suspension culture methods are spinner flasks and rotating-wall bioreactors [74,85]. Becker et al. [86] with the use of a rotating-wall vessel were able to form multicellular spheroids of LN1 cell line derived from a metastatic lesion of a patient with mixed miillerian tumor of the ovary. These spheroids kept in culture for more than 32 days showed a tendency to divide once they reached a certain characteristic cell size or density that could be correlated to the intrinsic cell line metastatic activity. In recent years, this technique has also been integrated with scaffold-based cell culture, through incubation in spinner flasks or bioreactors cell seeded or embedded into a porous polymeric matrix [87,88].

Working principles, advantages and disadvantages of ULA, HD and agitation-based methods for 3D tumor modeling are summarized in Table 2.

### 4.2. Scaffold-Based 3D Cell Culture

Further developments on 3D tumor cell culture have been possible thanks to rapid advancements in tissue engineering, an interdisciplinary field that applies the principles of engineering and life sciences for the development of 3D biological substitutes, which support cellular architecture by restoring, maintaining and mimicking the functions of native tissues [89]. In scaffold-based techniques, a physical support is used to mimic the natural structure of the ECM. Cells can be seeded directly onto this matrix, and the biological crosstalk between cells and scaffold is controlled by the material properties. In order to induce cell response and tissue growth, the scaffold should meet several requirements, as will be discussed in detail in the following sections [90]. It must be biocompatible and allow cells to adhere, proliferate, and migrate into its porous structure. Scaffold biodegradation kinetics should be tailored to allow cells to produce their own ECM and gradually replace the construct [91]. The design of a scaffold with an interconnected network of pores and a high surface porosity results in macro- and microstructural features that not only influence cells survival, signaling, growth and motility, but also play a fundamental role in effective diffusion of nutrients, gases, and metabolic waste products. Furthermore, the scaffold must have a pore size large enough to ensure cell migration and proliferation within the porous structure, and at the same time it must provide a sufficiently large surface area for adhesion of a critical number of cells. Finally, the scaffold should possess mechanical properties comparable to those exhibited by the target native tissue. In particular, various studies have well documented that the scaffold mechanical stiffness plays a fundamental role in maintaining the phenotype and aggressiveness of adhered tumor cells, influencing intercellular organization and the ability to form metastases [92].

Various studies carried out over the past years have highlighted that the employment of hydrogel scaffolds for OC modeling represents a powerful strategy to optimize in vitro tissue structural and functional features. In particular, a hydrogels matrix can provide a stiffness comparable to that of ovarian soft tissues, potentially resembling the structural and mechanotransductional role of natural ECM. A tailored hydrogel porous structure can, therefore, integrate a substrate directly influencing the morphology and behavior of the adhered cells, as well as a 3D microenvironment promoting fundamental cell–cell and cell–ECM interactions and incorporating key components of the ECM, such as proteins, growth factors, and nutrients [93,94].

## 5. Hydrogel-Based Cell Culture

Hydrogels are 3D hydrophilic polymeric networks that can absorb even more than 90% of water by volume while maintaining their structure, thanks to crosslinks among the macromolecular chains [95]. These swollen materials are soft and allow for free diffusion of nutrients, oxygen, and cell waste, making them suitable for biomedical applications. For example, they can be employed to support the regeneration of various tissues, such as cartilage, bone, and vascular tissues, especially thanks to their viscoelastic behavior providing mechanical compliance with the surrounding tissues when implanted [96]. Hydrogels are extensively used for 3D cell culture thanks to their tunable biochemical and biophysical properties, such as macromolecular architecture, porosity, shape, degradability, stiffness and other mechanical cues. This allows for mimicking in vitro the ECM properties of various pathological tissues, with the ultimate aim of studying their pathogenic process and drug response [97]. To date, many hydrogels are available on the market, such as MaxGel^™^, a human cell-cultured-derived ECM containing collagen, laminin, fibronectin, tenascin, elastin, proteoglycans and glycosaminoglycans [98,99], the HydroMatrix^™^ synthetic peptide that originates a nanofiber scaffold in response to changes in temperature or ionic strength [98,100], and the TrueGel3D platform, an animal origin-free polysaccharide hydrogel system validated for several applications, such as tumoral cyst and spheroid formation, as well as coculture of cancer and stroma cells [101,102]. These validated platforms provide 3D in vitro microenvironments more closely mimicking the in vivo counterparts, in comparison to conventional scaffold-free 3D spheroids [103].

### 5.1. Network Formation

Hydrogels for tumor modeling can be made from either natural, semisynthetic, or synthetic polymers (Table 3). In general, natural polymers are bioactive and promote cell adhesion, proliferation, and development, whereas conventional synthetic polymers lack cell-stimulating activity [104]. Natural polymers employed as hydrogels are usually proteins or ECM polysaccharide components (e.g., collagen, fibrin, and hyaluronic acid) or polysaccharides derived from other biological sources (e.g., chitosan and alginate). As a consequence, they are intrinsically biocompatible and bioactive thanks to the presence along their macromolecular backbone of functional groups recognized by cellular receptors, and promoting cell adhesion, cell-responsive degradation, and ECM remodeling. Nevertheless, modulating their mechanical properties can be difficult and batch-to-batch variability could be a relevant problem. By contrast, the structural and functional properties of synthetic polymer hydrogels (e.g., porosity, swelling degree, biodegradability, and mechanical strength) can be finely tuned to specific applications, minimizing the variability among different batches and optimizing experimental reproducibility. Furthermore, it is also possible to integrate specific peptides or sequences that help to properly mimic the native ECM and thus increase the bioactivity of synthetic polymers [105].

**Figure 4:**
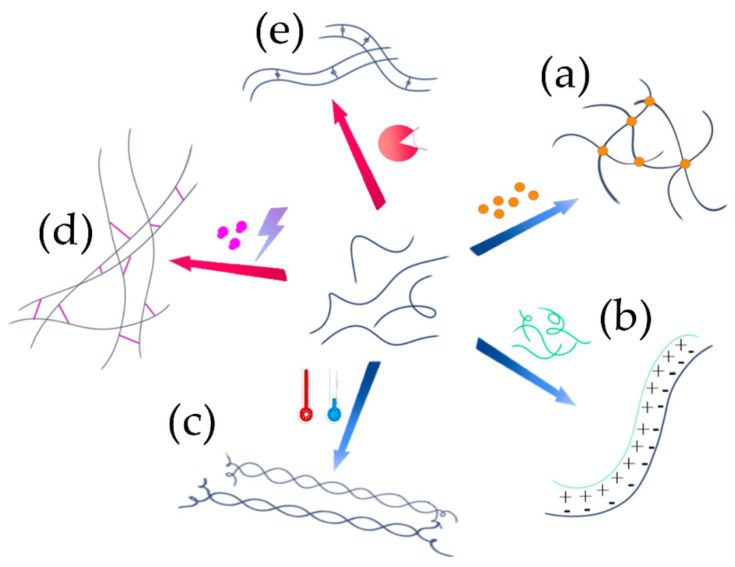
Graphical representation of hydrogel formation process through physical (blue arrows) or chemical (red arrows) interactions among macromolecular chains. (**a**) Ionic crosslinking, (**b**) polyelectrolyte complex (PEC) formation, (**c**) thermoresponsive gelling process, (**d**) photoactivated crosslinking, and (**e**) enzymatic crosslinking.

The hydrophilic polymer chain network can be formed via various crosslinking methods based on either physical or chemical strategies (Figure 4). Physically crosslinked hydrogels rely on usually reversible non-covalent interactions (e.g., ionic, electrostatic, and hydrophobic interactions, as well as hydrogen bonding) holding the chains together and providing the resulting network with elastic properties. As a consequence, a water swelling equilibrium is reached when the osmotic pressure within the network and the opposite elastic force of the crosslinked macromolecules balance each other [12]. Physical hydrogels (Figure 4a–c) are typically preferred over chemical ones for biomedical applications, thanks to the higher safety and lower cytotoxicity, related to the absence of unreacted chemical crosslinking agents [106]. On the other hand, chemically crosslinked hydrogels (Figure 4d,e) can provide better mechanical properties and superior physiological stability, resulting in a slower degradation rate, due to the irreversible crosslinking covalent bonds. Their formation typically involves free radical polymerization induced by light (e.g., photopolymerization), other chemical initiators, or crosslinking agents. Enzymatic-crosslinking is gaining increasing attention thanks to the possibility of forming covalent bonds without the employment of chemical mediators [106].

**Table 3:** Natural and synthetic polymers investigated for hydrogel tumor modeling.

Polymer(s)	Tumor Modeling	Cell Line	Ref.
*Natural source polymers*			
Chitosan	Breast cancer	4T1	[107]
Chitosan/Alginate	Glioblastoma	U87-MG U-118	[108]
	Prostate cancer	LNCaP C4-2 C4-2B TRAMP-C2	[109]
Chitosan/Hyaluronic acid	Glioblastoma	U-118 MG	[110]
Chitosan/Pectin	Colorectal cancer	HCT116	[111]
Chitosan/Silk Fibroin	Lung cancer	A549	[112]
Cellulose	Hepatic cancer	HepG2	[113]
	Cervical cancer	HeLa	[114]
	Breast cancer	MCF7 MDA-MB-231	[115]
Cellulose/Gelatin	Breast cancer	MDA-MB-231	[116]
Cellulose/Hyaluronic acid/ Gelatin	Glioblastoma	U251	[117]
Cellulose/Alginate/Lignin	Hepatic cancer	HepG2	[118]
Alginate	Breast cancer	MCF-7	[119]
	Neuroblastoma	SK-N-BE(2)	[120]
	Glioblastoma	U87-MG	[121]
	Hepatic cancer	HepG2	[122]
Alginate/Gelatin	Breast cancer	MDA-MB-231	[123]
	Colorectal cancer	HCT116	[124]
Agarose	Breast cancer	MCF-7	[125]
	Cervical cancer	HeLa	[126]
	Glioblastoma	U251	[127]
	Lung cancer	A549	[128]
Agarose/Collagen	Breast cancer	MCF-7 MDA-MB-361 MDA-MB-231	[129]
	Glioblastoma	U373-MG	[130]
Hyaluronic acid derivatives	Breast cancer	MCF-7	[131]
	Prostate cancer	LNCaP	[132]
	Glioblastoma	U373-MG U87-MG	[133]
Hyaluronic acid/Alginate	Prostate cancer	PC3 DU145	[134]
Collagen	Breast cancer	MDA-MB-231	[135]
	Glioblastoma	U87	[136]
	Hepatic cancer	HepG2	[137]
Collagen/Alginate	Breast cancer	MDA-MB-231	[138]
Gelatin	Breast cancer	MCF-7	[139]
*Synthetic polymers*			
Poly(ethylene glycol) (PEG)	Breast cancer	MDA-MB-231	[140]
	Glioblastoma	U251	[141]
	Pheochromocytoma	PC-12	[142]
Poly(vinyl alcohol) (PVA)	Glioblastoma	LN229 U87-MG	[143]
	Pancreatic cancer	PaTu 8988t	[144]
*Hybrid polymeric materials*			
Polycaprolactone/Cellulose/ Gelatin	Glioblastoma	U251-MG	[145]
PEG/Chitosan	Breast cancer	MMC	[146]
PEG/Collagen	Hepatic cancer	HepG2	[147]
PEG/Fibrinogen	Breast cancer	MDA-MB-231	[148]
PEG/Gelatin	Fibrosarcoma	HT1080	[149]
PEG/Silk fibroin	Lung cancer	A549	[150]
Poly(methyl vinyl ether-*alt*-maleic acid) (PMVE-*alt*-MA)/Hyaluronic acid	Hepatic cancer	HepG2	[151]
Poly(Nɛ-acryloyl l-lysine)/ Hyaluronic acid	Breast cancer	MCF-7	[152]
PVA/Cellulose	Breast cancer	MDA-MB-231	[153]
PVA/Gelatin	Hepatic cancer	HepG2	[154]

**Electrically charged polymers**, or polymers whose net charge can be revealed in acidic or alkali solutions, are crosslinked with ions or other macromolecular compounds of opposite sign, hence forming polyelectrolyte complexes (PECs) (Figure 4b). For example, alginate is negatively charged in an aqueous environment due to the presence of carboxyl groups in its repeating unit; therefore, it can either interact with divalent cations (e.g., Ca^2+^ and Mg^2+^) in a conformation known as the egg-box model, or with another positively charged polyelectrolyte such as chitosan. The resulting network is insoluble in aqueous media because of the shielding of the charged groups [155]. One of the main advantages of PEC hydrogels is their self-healing ability: once the hydrogel is broken upon mechanical stress application, the physical network can be recovered autonomously and the structural damage repaired, once the load is released. Nevertheless, their mechanical strength is typically limited [106] and they can dissolve in an aqueous environment under certain conditions, such as when in contact with chelating agents [155].

**Hydrogelators** are another class of charged molecules that can form physical hydrogels by self-assembling into 3D supramolecular networks. Among them, self-assembling peptides that can autonomously organize in water into ordered nanofibers and further into scaffolds are particularly attractive for biomedical applications [156]. These short oligopeptides can have different secondary structures, such as α-helix, β-sheet, or random coil. Depending on their sequence, charge distribution and chirality, they are characterized by inter- and intramolecular weak specific interactions (e.g., hydrogen bonds and π–π stacking) or strong non-specific interactions (e.g., electrostatic interactions) [156]. An example is RADA-16, an ionic self-complementary peptide made of three amino acid residues (R-arginine, A-alanine, and D-aspartic acid).

**Thermoresponsive polymers** represent another group of macromolecules employed to prepare physically crosslinked hydrogels, which can be divided into two classes depending on the gelling process (Figure 4c). Polymers that undergo a sol–gel transition upon heating have a lower critical solution temperature (LCST), below which they become miscible with their solvent. For example, below 30 °C, elastin is solvated by water molecules, whereas upon heating it undergoes a sol–gel transition by which the chains fold to form nanoparticles that can entrap bioactive compounds for drug delivery strategies [157]. The main driving force of this process is the entropy gained through chains dehydration [158]. Indeed, at low temperatures, polymer molecules are solubilized thanks to hydrogen bonds with water molecules, resulting in a one-phase system; upon increasing the temperature, the hydrogen bonds are weakened, and the polymer chains become desolvated. A different chain conformation is then assumed to minimize the macromolecular surface exposed to water, for example from random coil to helix or from coil to a globular conformation [158]. On the other hand, the majority of natural thermoresponsive polymers (e.g., agarose and gelatin) have an upper critical solution temperature (UCST), above which they are water-soluble. In this case, the gelling process takes place by cooling and it is driven by a change in enthalpy due to the increasing hydrophobic properties of the polymer [159]. Due to the physical interactions keeping together the network, thermally crosslinked hydrogels can be dissolved by reversing the temperature change [155].

**Photo-crosslinking** is one of the most exploited approaches to chemically crosslinked hydrogel fabrication (Figure 4d). Hydrogels formed by light irradiation need the presence of photo-initiator compounds that, after irradiation, form free radicals able to react with unsaturated groups present in the polymer backbone, with the resulting formation of intermolecular covalent bonds. The wavelength typically employed is in the UV region, e.g., centered at around 365 nm, whose intensity is tolerated by most cell types if exposed for less than a few minutes, hence providing a potential tool for cell encapsulation. The advantages of photo-crosslinked hydrogels are their rapid network formation under mild conditions and the modulability of their mechanical properties by controlling the crosslinking reaction conditions. Their main disadvantage is the possible presence of unreacted crosslinking molecules that can induce an immunogenic response [106]. To overcome this drawback, tailored cytocompatible photo-initiators have been developed to fabricate gels for biomedical applications [160].

**The sol–gel method** can be used to obtain solvated gel networks through polymerization of small molecular precursors [161,162]. The solvent is not necessarily water in the first place, but once the gel network is formed, the solvent can be replaced with water. For instance, supercritical fluid dried aerogels can be fabricated through a wide range of molecular precursors using the sol–gel technology and tailored drying methods (see Section 5.3) [163].

**Enzymatic crosslinking** is an emerging biocompatible strategy to create covalent bonds among macromolecular chains (Figure 4e). This technique is inspired by natural reactions occurring in our body, such as those involved in covalent crosslinkages that stabilize collagen and elastin and mediated by lysyl oxidase, a key component in the formation and remodeling of ECM [164]. The main advantage of enzymatically crosslinked hydrogels is the mild reaction conditions, occurring in an aqueous medium, at neutral pH, and physiological temperature; indeed, most of the enzymes employed for this purpose catalyze reactions naturally occurring in our body, and their activity can be modulated to control the gelling process. Therefore, this approach is investigated to develop injectable in-situ-forming hydrogels with biomimetic mechanical and swelling properties. Additionally, thanks to the enzyme-substrate specificity and absence of photo-initiators or other chemically reactive molecules, cytotoxic reactions are typically avoided [165].

### 5.2. Properties

**Figure 5:**
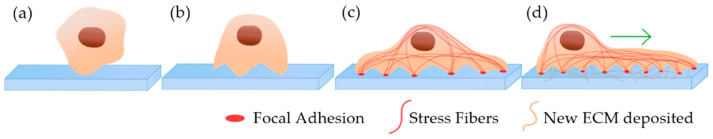
The cell adhesion process on scaffold surface. (**a**) The cell comes in contact with scaffold surface and loosely attaches onto the substrate, (**b**) the cell starts to flatten, (**c**) the cell spread its membrane and form focal adhesions that connect the cell securely on the scaffold surface with intracellular actin filaments (stress fibers) through integrin, and (**d**) the cell begins to migrate to the scaffold surface generating new EMC (the green arrow indicates migration direction).

Biocompatibility and bioactivity are fundamental scaffold properties. Appropriate cellular adhesion is crucial for the success of the cell proliferation process and the resulting formation in vitro of a tumoral tissue, which takes place through different stages: (i) cell attachment, (ii) cell spreading, (iii) focal adhesion between cells and scaffold surface, and (iv) migration onto its surface and through its porous structure while laying down new ECM (Figure 5) [166].

**Biocompatibility** has been formally defined in 1987 as “the ability of a biomaterial to perform with an appropriate host response in the specific application”. Any material selected to develop scaffolds must be evaluated to determine the unwanted damage or side effects that could generate to the host. The three main responses that should be considered are: inflammation, wound healing, and immunological/immunotoxicity reaction [167]. Natural polymer-derived hydrogels intrinsically present bioactive sequences within their structure, whereas synthetic polymers in most cases need to be further modified to stimulate an appropriate cell response and induce the required biological interactions in 3D cell culture. For example, cell-binding motifs (e.g., RGD sequence from fibronectin, IKVAV sequence from laminin, and GFOGER sequence from collagen) or growth factors can be employed to functionalize the scaffold and enhance its recruitment of specific cells, with the overall result of providing an inductive environment that stimulates cell differentiation and tissue regeneration. These bioactive peptides will constitute an integral part of the biohybrid network and can be covalently bound to the backbone and released as the cells degrade the hydrogel, or can be entrapped within the porous structure of the polymeric construct [105].

**Biodegradability** is another fundamental requirement, especially when hydrogels are employed in tissue regeneration and not designed as permanent implants; in this case, cells need to colonize the scaffold, produce their own ECM, and gradually replace the implanted construct while it is eroding. However, in the case of in vitro tissue modeling, scaffold’s bioerosion tailored to tissue development is not often achievable within the experimental time scale (typically few weeks), and as a consequence polymer complete biodegradation becomes non-essential. In any case, the scaffold’s bioerosion rate must be adapted to a specific application to match cells’ ability to synthesize new ECM while, at the same time, providing appropriate structural support. Scaffold’s bioerosion can happen through various mechanisms, such as polymer dissolution, hydrolysis or enzymatic catalysis. In particular, hydrogels made of polymers naturally present in the ECM (e.g., collagen, fibrin, elastin, and hyaluronic acid) are degraded by cells’ proteases, whose catalytic action can be increased, especially towards synthetic polymers, by including enzyme-sensitive peptide crosslinkers within the macromolecular backbone [168]. Nevertheless, it is also possible to control the hydrolysis rate by introducing in the macromolecular network hydrolytically degradable crosslinkers, such as ester linkages, providing a time-dependent degradation [168].The degradation products should be non-toxic, non-immunogenic, and excreted from the body without interfering with cellular metabolism. In addition, a correlation has been found between degradation and cellular differentiation, for instance into an osteogenic lineage, underling the importance of matrix remodeling in cellular behaviors [169]. Scaffold-remodeling as an important feature that stimulates cellular response can also be obtained through network debonding in physically crosslinked hydrogels, as well as through stress relaxation in ionically crosslinked hydrogels [170]. As examples, time-dependent stress changes (stress relaxation) were demonstrated to increase fibroblast spreading [171] and osteogenic differentiation [172] by enhancing the rearrangement of focal adhesions, as well as to significantly improve the production of ECM by chondrocytes [173].

**The mechanical properties** of the adhesion substrate strongly influence cell morphology and signaling pathways. Indeed, cells receive mechanical feedback from the substrate onto which they adhere. After cell seeding, cells interact with the substrate through transmembrane glycoproteins called integrins. As cells bind to the ECM, integrins begin to clump, leading to the recruitment of structural and signaling proteins to form so-called focal adhesions at the integrin clustering site. The formation of focal adhesions requires the application of cell mechanical forces to these adhesion points. In general, if a substrate is stiff, cells generate large forces that lead to the formation of mature focal adhesions and a highly organized cytoskeleton with abundant stress fibers. On the other hand, a soft substrate cannot provide sufficient strength to balance large forces generated by the cells. Therefore, on soft substrates, the cells do not develop abundant stress fibers and generate less forces. Changes in the organization of the cell cytoskeleton are important because the cytoskeleton is involved in many signaling pathways that transfer mechanical feedback into chemical responses. Furthermore, the cytoskeleton also determines the shape of a cell, which in turn is intimately related to cellular behavior [174]. Hydrogel mechanical properties are affected by various factors, such as the macromolecular structure of the polymer, the crosslinking strategy and density, the gelling reaction, the polymer degradation rate, and the degree of water uptake. For example, natural polymer hydrogels typically display poor mechanical properties that can be enhanced by fabricating composite hydrogels, reinforced with nanofibers, or interpenetrated polymer networks (IPNs) [175,176]. Hydrogels viscoelastic behavior is the result of the high swelling degree and the polymer network mechanical properties [170]. Overall, the higher the swelling degree, the lower the mechanical stiffness would be. Therefore it is fundamental to find a right balance to properly mimic the native ECM, especially since matrix stiffness is considered a key parameter in tumor development [67]. For example, in the case of epithelial OC modeling, a stiffer matrix promotes cell spreading, focal adhesion formation, random cell migration and the disaggregation of multicellular spheroids, a typical behavior associated with the metastatic process [177]. Nevertheless, an enhanced malignant phenotype of metastatic OC cells characterized by high proliferation rate and grater chemo-resistance has also been reported in softer substrates [22]. From these controversial results, the need to further develop and optimize 3D OC culture models emerges also in consideration of the genetic complexity, diverse pathology, and the unique mechanisms underlying metastasis of this kind of tumor.

**Hydrogels’ morphology and porosity** are other fundamental characteristics not only to guarantee the diffusion of medium, nutrients, gases, cellular waste products and degradation by-products throughout the scaffold, but also to ensure cellular penetration, proliferation, and migration [178]. In particular, microporosity is fundamental for cell–ECM interactions and capillary ingrowth, especially in tissue engineering applications, where microporosity is relevant to nutrient and gases supply, as well as cell metabolism waste removal. Scaffold porosity should be tailored to the target tissue, with a critical range of pore size depending on the cell type and the ECM synthesis rate [90]. For example, the minimum pore size required for bone tissue regeneration is considered to be about 100 μm on the basis of cell size, migration conditions, and nutrients transport; however, pore sizes larger than 300 μm are recommended to improve new bone formation and to develop a net of capillaries [179]. Nevertheless, porosity is inversely related to mechanical stiffness; thus, it is important to properly balance these two properties in order to maintain structural stability, while providing adequate and efficient perfusion of nutrients and gasses, especially since the diffusion capacity of oxygen is limited to a distance of 100–200 μm [180]. The available surface area of hydrogels can be optimized by engineering their porous structure through various methods, as discussed in the next section relevant to their fabrication. Furthermore, thanks to new microengineering techniques (e.g., micromolding, 3D bioprinting, photolithography, and stereolithography) it has become possible to deliver hydrogels with not only defined macropore size and shape, but also microfluidic channels that mimic the tissue microvascularization [181].

### 5.3. Fabrication

**Figure 6:**
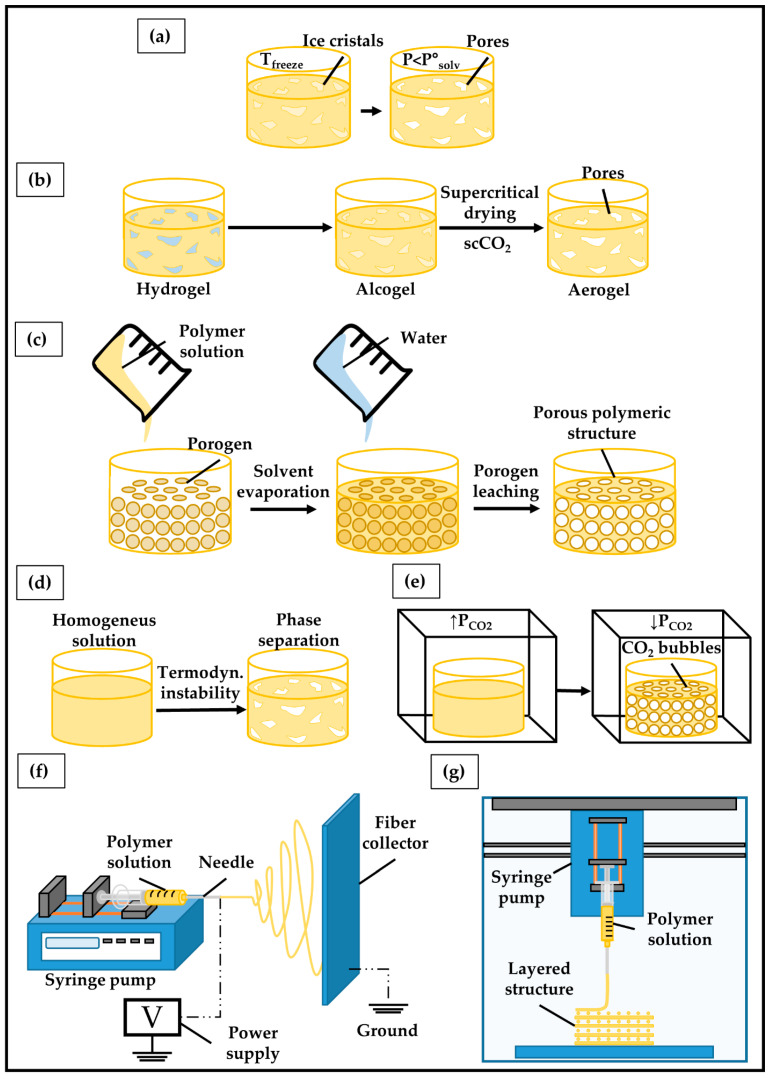
Schematic representation of scaffold fabrication techniques. (**a**) Freeze-drying, (**b**) supercritical drying, (**c**) solvent casting, (**d**) phase separation, (**e**) gas foaming, (**f**) electrospinning, and (**g**) additive manufacturing.

Tissue scaffolds have been fabricated by different techniques, including freeze-drying, supercritical drying, solvent casting, gas foaming, phase separation and electrospinning (Figure 6) [182].

**Freeze-drying** procedure is divided into two distinct phases. Firstly, a polymer solution or dispersion is cooled down to a temperature at which all the material is in a frozen state, resulting in the formation of solvent ice crystals. Subsequently, a pressure lower than the equilibrium vapor pressure of the frozen solvent below its triple point (P_solv_) is applied to induce sublimation [183]. Removal solvent ice crystals leads to the formations of a highly porous sponge (Figure 6a).

**Supercritical drying** methods are based on wet-gel drying through the use of a supercritical fluid, typically scCO_2_, which selectively extracts a liquid from the porous structure [184,185]. For instance, a hydrogel can be formed starting from an aqueous colloidal suspension of precursors (sol) through hydrolysis/polycondensation reactions. Because of its low solubility in scCO_2_, water in the gel structure is replaced with ethanol or methanol to form an alcogel, which is finally dried by scCO_2_ (Figure 6b). This approach is widely used to fabricate aerogels with a well-defined porous structure, by minimizing the pore collapse and material cracking/shrinkage phenomena, which can occur during freeze drying.

**The solvent casting** technique involves dissolving the polymer into a suitable solvent and casting the resulting solution into a mold holding porous particles (e.g., sodium chloride or paraffine spheres) [186]. After the polymer solution has been poured, the solvent is allowed to evaporate leaving behind a polymer matrix incorporating salt particles. Subsequently, the solid structure is immersed in a bath of a solvent selectively leaching out the porogen to obtain a porous structure (Figure 6c).

**Gas foaming** is a solvent-free technique for the fabrication of porous materials through the generation of gas bubbles within a polymer matrix [187]. A molded polymer kept at a temperature higher than its glass transition temperature is exposed to a high-pressure gas to allow its saturation into the solid matrix. The subsequent decrease in gas pressure causes the nucleation of gas bubbles, with the resulting formation of a porous structure (Figure 6d).

**Phase separation** processes are based on a thermodynamic instability induced in a homogeneous polymer solution that, as a consequence, separates into two or more phases to lower the total free energy [188]. The most exploited approaches for porous scaffold fabrication rely on a thermally or non-solvent-induced phase separation. Under tailored kinetic and thermodynamic conditions, the polymer solution can be induced to separate into a polymer-lean phase dispersed into a polymer-rich phase, finally resulting in a highly porous polymeric network (Figure 6e).

**The electrospinning** process involves the formation of a polymeric fluid jet by establishing an electric field between a needle of a syringe containing a polymer solution or suspension and a grounded device, acting as fibers collector [189]. During its travelling towards the collector, the jet is thinned due to electric elongational forces and the solvent evaporates, resulting in the formation of nano/microfibers continuously deposited onto the counterelectrode (Figure 6f).

**Figure 7:**
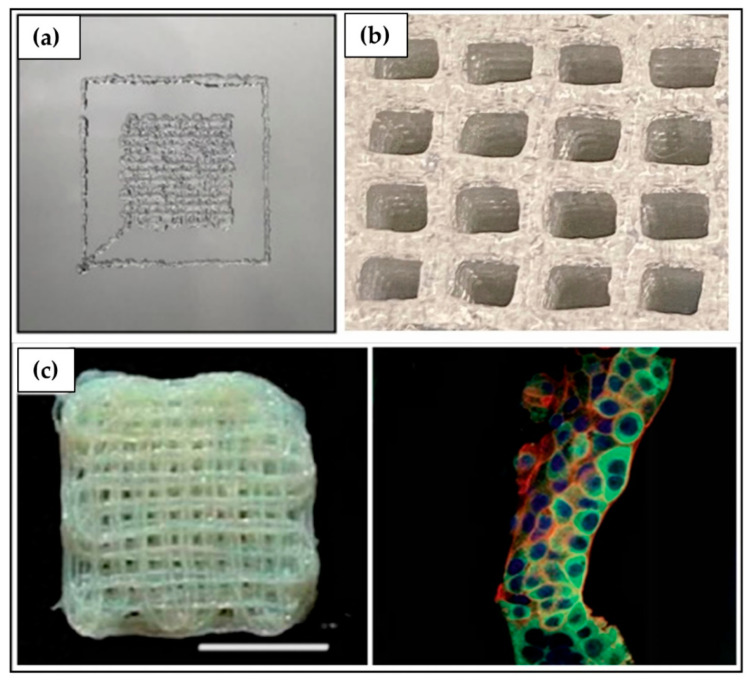
Hydrogel-based scaffolds by additive manufacturing (AM). (**a**) Top view of 3D bio-printed construct based on GelMA [192], (**b**) photographic image of ten-layer printed scaffold with Ink H4-RGD [193] and, (**c**) representative photograph of a chitosan/γ-PGA PEC hydrogel and representative confocal laser scanning microscopy micrograph of BxPC-3 cells grown on the PEC hydrogel [97].

The above-described fabrication approaches do not guarantee a high reproducibility and control of the parameters that define the scaffold shape and porous macro- and microstructure. Despite this, they are still widely employed thanks to the low production costs and limited complexity of the required equipment [190]. The growing interest in 3D cell culture and customized scaffolds has increased the need for new technologies that make it possible to overcome these disadvantages. In this context, additive manufacturing (AM) (Figure 6g), also referred to as 3D printing, comprises a set of techniques based on a computer-aided design and manufacturing process for the layer-upon-layer fabrication of a 3D scaffold with a high degree of reproducibility and advanced control over external shape, as well as pore size, geometry, and distribution [190,191]. In addition, modern Bioprinting techniques involve processing a cell culture formulation containing a polymeric material and suspended cells to directly fabricate cell-laden hydrogels. As an example, a recent study by Kim et al. [192] described the fabrication of gelatin methacryloyl (GelMA)-based hydrogels embedding bladder cancer cells that were subsequently submitted to UV photocrosslinking. The resulting tissue construct displayed a well-ordered and porous 3D structure, which allowed for higher cell proliferation, interaction, and chemotherapeutics sensitivity than 2D cell culture used as a reference (Figure 7a). Gebeyehu et al. [193] developed a stable and ready-to-use polysaccharide-based bioink, which could be used to form spheroids of non-small-cell lung cancer cells within 7 days of culture (Figure 7b). In vitro cytotoxicity studies conducted on these 3D printed spheroids showed greater resistance to doxorubicin, docetaxel, and erlotinib compared to 2D monolayers, suggesting their suitability for high throughput screening of anti-cancer drugs. Chiellini et al. [97] reported the possibility of processing by computer-aided wet-spinning a chitosan/poly(γ-glutamic acid) (γ-PGA) PEC [194] for the production of porous hydrogels that allowed for the long-term in vitro culture of pancreatic ductal adenocarcinoma cells (Figure 7c).

## 6. Polymers for 3D Ovarian Cancer (OC) Modeling

A wide range of polymeric hydrogels have been used to support 3D ovarian tumor cell culture. In particular, hydrogels from polymers of natural origin, such as polysaccharides (e.g., chitosan, cellulose, alginate or agarose) and proteins (e.g., collagen or gelatin), or synthetically polymerized, such as RADA16-I and poly(ethylene glycol) (PEG) have been investigated as scaffolds for OC cells [195]. Although natural biomaterials exhibit high biocompatibility, including intrinsic biological signaling with a surface chemistry that promotes cell adhesion, reactive cell degradation and remodeling, they have some limitations due to immunogenicity and batch-to-batch variability, which decrease experimental reproducibility. On the other hand, synthetic biomaterials allow for scaffold production with controlled properties, such as stiffness, degradation rate, and porous structure, but they lack in biological activity and cell stimulation [196].

Pioneering studies on scaffold-based techniques for in vitro 3D OC modeling are overviewed in the next section by highlighting the polymeric materials employed for hydrogel development, as well as relevant tumor biology and drug screening results (Table 4).

### 6.1. Polysaccharides

#### 6.1.1. Chitosan

**Figure 8:**
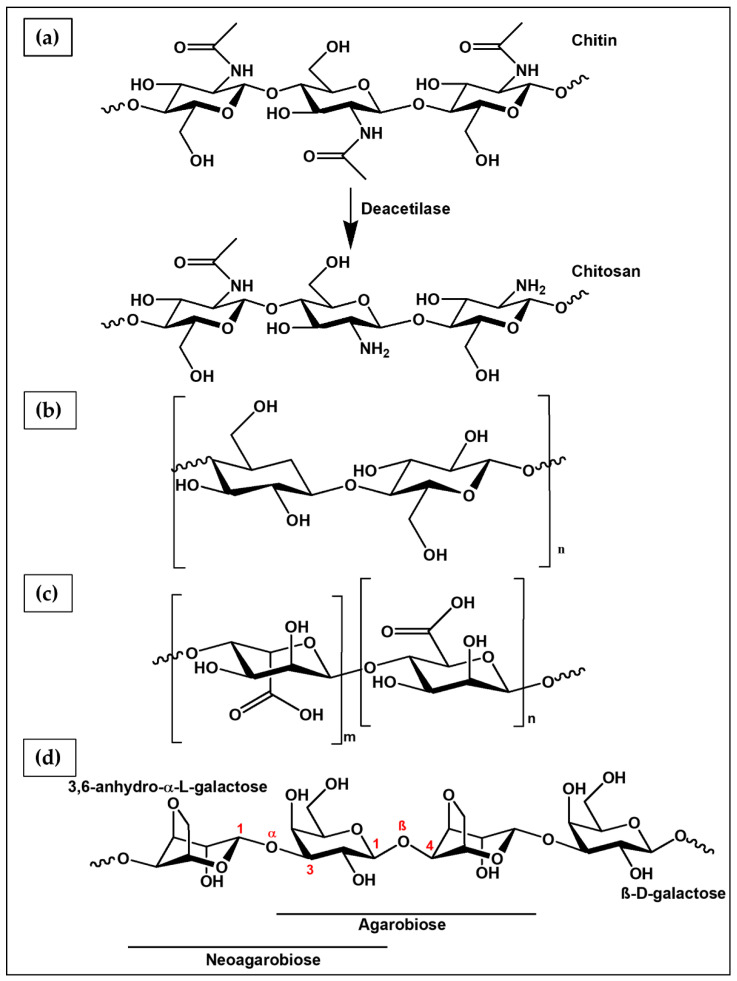
Chemical structure of polysaccharides investigated for hydrogel-based scaffolds in OC modeling. (**a**) Chitosan obtained by chitin denaturation, (**b**) cellulose, (**c**) alginate, and (**d**) agarose.

Chitosan (Figure 8a) is obtained from its precursor chitin by alkaline hydrolysis of the acetate groups. Chitin is the most abundant polysaccharide in nature after cellulose, found in the exoskeleton of crustaceans (e.g., shell of shrimp, lobster, krill and crab), insects, and some fungi [96]. Chitin is an unbranched polymer formed by repeating units of *N*-acetyl-D-glucosamine, and when the deacetylation process leads to the removal of more than 50% of the acetylated functional groups, it is converted into chitosan [155]. Chitosan is a linear copolymer of β-(1–4) linked 2-acetamido-2-deoxy-β-D-glucopyranose and 2-amino-2-deoxy- β-D-glycopyranose units. The hydrolysis process of the N-acetyl-D-glucosamine units occurs mainly by treating chitin with an aqueous solution of NaOH (40–45% *w*/*v*) at 90–120 °C for 4–5 h. Depending on the conditions used in this process, the deacetylation degree (DD) of chitosan changes, with a significant effect on polymer chemical, physical, and biological properties, such as solubility, crystallinity, biodegradability, viscosity, and biocompatibility [96,210]. Chitosan is commercially available in various percentages of DD, commonly classified as low (55–70%), medium (70–85%), high (85–95%), and ultra-high (>95%) degree [211]. The insolubility of chitin in most organic solvents is due to the presence of acetyl groups in its repeating units, while chitosan results to be a cationic polyelectrolyte soluble in acidic aqueous solutions (Ph < 6) because of the protonation of the amino groups (-NH_3_^+^), which have a PKa value of 6.3 [96]. As a polycation, chitosan can simply behave as a hydrogel through the establishment of ionic interactions with low molecular weight molecules that have opposite charge (e.g., β-glycerophosphate and tripolyphosphate salt) or polyanions (e.g., alginate, pectin, elastin and DNA) [212]. A photo-cured glycol chitosan hydrogel containing paclitaxel-complexed β-cyclodextrin was recently investigated for OC therapy using a tumor-bearing mouse model [213].

**Figure 9:**
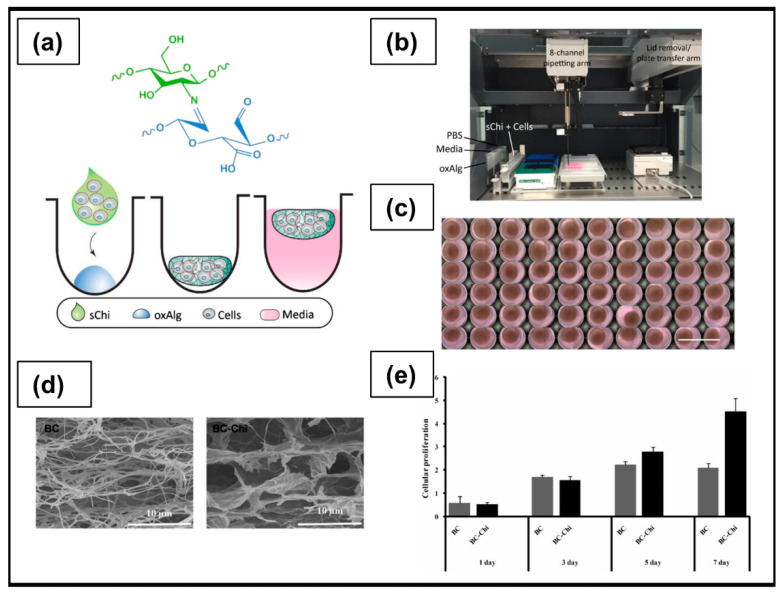
Chitosan and cellulose hydrogels. (**a**) Chemical structure of sChi (green)-oxAlg (blue) unit, and schematic representation of cell seeding process in U-bottom 96-well plates; (**b**) setup for automated seeding of cells via Tecan Freedom EVO liquid handling station; (**c**) cell-seeded scaffolds at day 1 (scale bar = 10 mm) [197]. (**d**) SEM morphology of BC and BC-Chi scaffolds; (**e**) A2780 cell proliferation on BC and BC-Chi scaffolds for 1, 3, 5, and 7 days [198].

Kletzmayr et al. [197] developed a chemically modified alginate derivative through an oxidation process (oxAlg) to form hydrogels via chemical crosslinking with N-succinyl-chitosan (sChi). The developed hydrogel, obtainable inside the 96-well plates classically used in 2D cell culture studies, was able to support the growth of ovarian, lung, and prostate cancer cells, and provided a fast and reliable new platform for high-throughput drug screening (Figure 9a–c).

#### 6.1.2. Cellulose

Cellulose (Figure 8b) is the most abundant biodegradable polymer derived from biomass. Its primary source is the lignocellulosic material existing in plants; in particular, wood and cotton represent the main sources [214]. Bacterial cellulose (BC) is synthesized extracellularly mainly by *Acetobacter xylinum* species, with the main advantage of a higher purity degree than plant cellulose, which often contains vegetal residues, such as lignin, pectin, and hemicellulose [215,216]. Thanks to unique characteristics in terms of biodegradability, biocompatibility, low production cost, abundance, and excellent mechanical properties, cellulose is widely used in numerous medical applications, including wound healing, artificial blood vessels, drug delivery, dental grafting, and bone tissue engineering [217,218]. From a chemical point of view, it is composed of a linear chain of repeating units (several hundreds to many thousands) of sugar D-glucose, held together by bonds β(1→4). The bond is formed between the C-1 carbon atoms of one glucopyranose ring and the C-4 of the next, in a condensation reaction that leads to the elimination of a water molecule originated from the combination between the H and –OH group [219]. Thanks to the presence of numerous hydroxyl groups along the polysaccharide chain, capable of establishing intermolecular hydrogen bonds, cellulose-based hydrogels can be developed through the formation of physical crosslinking [214,220]. Hyaluronic acid-blended carboxymethyl cellulose films are clinically used as anti-adhesion membrane during abdominal surgeries for OC [221]. According to a study performed by Ul-Islam et al. [198], BC porous hydrogels obtained by freeze drying (Figure 9d) could be used as 3D scaffolds for OC growth. Furthermore, the single-step formation of a PEC through interactions between the negative charges present in the BC structure and the positive ones of chitosan (BC-Chi) led to a significant increase, compared to pure BC, of A2780 OC cells proliferation up to 7 days (Figure 9e). Confocal laser scanning micrograph analysis highlighted cell migration throughout the scaffold; the strong cellular adhesion to the BC-Chi scaffold surface and the formation of very small cell masses were supported by the down-regulation of the Notch receptor, responsible for cell–cell interaction and cell aggregation.

#### 6.1.3. Alginate

Alginate (Figure 8c) is mainly obtained by extraction from the cell wall of three types of marine brown algae (i.e., *Laminaria hyperborea*, *Ascophyllum nodosum,* and *Macrocystis pyrifera*), in which it constitutes up to 40% of the dry weight [222]. Although alginate is commercially derived from algal sources, in recent years an alternative type of production has been explored by means of bacterial fermentation to obtain alginate batches with more defined physical properties and chemical structure. Bacterial alginate is synthetized from *Azotobacter vinelandii* and several *Pseudomonas* species [223]. Alginate is a linear, polyanionic polysaccharide formed by both (1,4)-linked β-D mannuronic and α-L-guluronic acids blocks, namely, M-block and G-block, respectively [224]. Depending on the alginate extraction source, the environmental conditions, and the climatic changes to which the source was subjected, the sequence and relative ratio between the G- and M-blocks are not constant along the polymer chain. This generates a huge variability in the chemical-physical behavior of the material, as a consequence of the poor batch-to-batch reproducibility in composition, molecular weight, and residue sequence [191]. For instance, various studies have well documented that the mechanical properties of alginate-based gels are strongly influenced by the ratio of G- and M-blocks present in the polymer chain [225]. Alginate can form hydrogels through ionic interaction between carboxyl groups and divalent cations (the most used is Ca^2+^ thanks to the relevant mild reaction conditions and low toxicity). G-blocks are able to establish ionic bonds with cations, but recent studies have observed that alternating MG-blocks can also participate to form a weak junction [226]. In particular, alginate polymers containing a large number of G-block repeating units generate hydrogels with high mechanical strength. In contrast, alginate having a predominance of M-blocks results in flexible and soft hydrogels [12,224]. Physical-chemical properties of alginate hydrogels can be tailored depending on the nature of cross-linking bonds, the density of the intermolecular interactions, and the polymer molecular weight. Thanks to this variability, alginate is used in various biomedical applications, including wound healing, drug delivery and tissue engineering [222].

Alginate beads obtained by ionic crosslinking with a divalent cation have been widely used to maintain in vitro various types of cancer tissues. King et al. [227] further developed this technique for the inclusion within the beads of fragments of ovarian and oviductal organ pieces that could be cultured in vitro for up to two weeks. If necessary, the hydrogel could be degraded using alginate lyase, an enzyme that does not cause the degradation of the encapsulated tissue, allowing for cell recovery for subsequent morphological and molecular analysis. In another study, Marella et al. [87] encapsulated OC 3D SKOV-3 cells into alginate beads and cultured the resulting tissue engineered constructs inside a fluid-dynamic bioreactor (MIVO^®^) device able to mimic the native capillary microfluidics feeding the tumor. Dynamic cell culture inside the MIVO^®^ fluidic device showed divergent results after cisplatin treatment compared to static cell culture. After seven days of culture in the presence of cisplatin 10 μM under dynamic conditions, cell viability was 50%, significantly reduced compared to that obtained under static conditions (above 80%). These results were corroborated by immunostaining micrograph analysis highlighting that Ki67-positive cells (proliferative cells) were well evident after drug treatment in a static environment, especially in the inner part of the hydrogel, while caspase-positive cells (apoptotic cells) were predominantly present in the case of dynamic culture. In addition, it is interesting to note that the outcome of the efficacy of the drug tested in the xenograft model was comparable with that carried out in the MIVO^®^ device. Hydrogels can be also formed by means of a double-network composed of poly(ethylene glycol) (PEG)-covalently crosslinked poly(methyl vinyl ether-*alt*-maleic acid) (PMVE-alt-MA) and alginate ionically crosslinked with Fe^3+^. This type of hydrogel was able to support the long-term growth, proliferation, and spheroid formation of SKOV3 cells, with epithelial–mesenchymal transition (EMT), interleukin-6 (IL-6) and Wnt pathways overregulation, affecting the invasiveness of OC [201].

#### 6.1.4. Agarose

Agarose (Figure 8d) and agaropectin are the main components of agar. Agarose is a linear polysaccharide obtained by extraction from the cell walls of red algae belonging to the *Rhodophyceae* class [155]. Common procedures adopted to purify these two polysaccharides involve the use of quaternary ammonium salts to precipitate agaropectin, and PEG to precipitate agarose [228,229]. Agarose is a copolymer composed of two alternating monosaccharide units, the β-D-galactose and 3,6-anhydro-α-L-galactose, linked by glycosidic bonds (β 1–4) (between β-D-galactose and 3,6-anhydro-α-L-galactose, also called agarobiose) and (α 1–3) (between 3,6-anhydro-α-L-galactose and β-D-galactose, constituting the neoagarobose) [229,230]. Agarose aqueous solutions undergo a sol–gel transition upon cooling at a certain temperature depending on the concentration, molecular weight, and chemical structure [231]. The gelation process is reversible and takes place in three phases: induction, gelation, and quasi-equilibrium. Initially, above the sol–gel temperature, the agarose shows a random coil conformation in solution. Upon cooling, it assumes a double helix shape and finally forms a macroreticular network stabilizing the structure [232]. Agarose hydrogels have been widely used in wound and foam dressing, as drug delivery systems, and in tissue engineering as stiff matrices for cartilage studies [232,233].

Agarose is one of the polysaccharides used as a coating for the preparation of ULA plates for the generation of cellular spheroids. Agarose coating is a simple, fast procedure, not requiring the use of complex equipment and is among the less expensive solutions available on the market for ULA systems [234]. Because of these advantages and its innate properties of generating a soft support that imitates in a more representative way the biological tissues, agarose coating has been used to generate spheroids of OC cell lines, as well as of primary culture isolated from patients with stage III or IV ovarian papillary serous cystadenocarcinoma [235,236].

**Figure 10:**
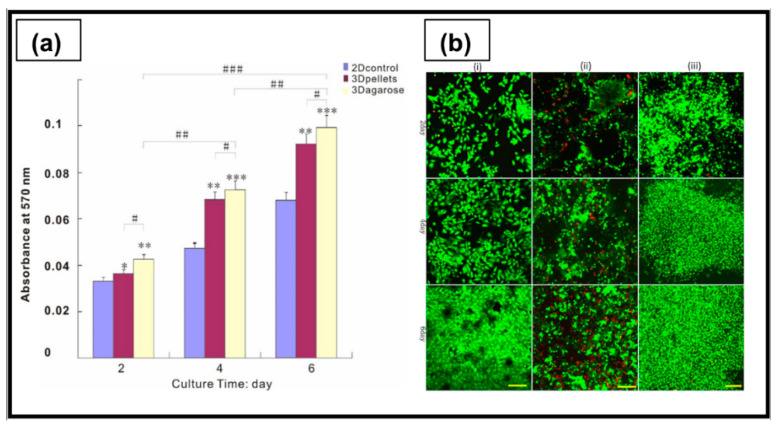
Agarose hydrogel. (**a**) SKOV3 cell proliferation in 3D and 2D cultures. */#, **/##, and ***/### denote *p* < 0.05, *p* < 0.01, and *p* < 0.001, respectively. (**b**) Live/dead cell assay: (i) 2D control, (ii) 3D control, and (iii) 3D agarose. Scale bar is 100 µm [199].

In a recent study, agarose-based hydrogels, obtained by thermal cross-linking at 90 °C, were investigated for anticancer drug screening. Agarose-based hydrogels sustained higher proliferation of ovarian tumor SKOV3 cells, compared to reference 2D cell culture, with a rounded cell shape and homogeneous cell distribution inside the constructs (Figure 10). Moreover, cells grown on the agarose hydrogels were able to maintain typical aggressiveness of OC, demonstrated by the upregulated expression of pro-angiogenic and hypoxic factors (VEGF-A and HIF-1α), compared to 2D cell culture [199].

### 6.2. Proteins

#### 6.2.1. Collagen

**Figure 11:**
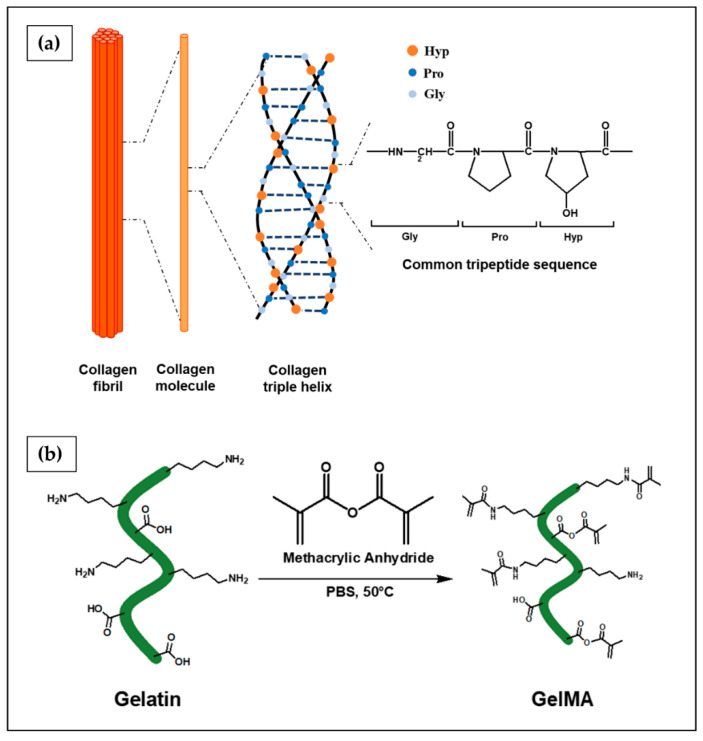
Chemical structure of proteins investigated for hydrogel-based scaffolds in OC modeling. (**a**) Triple chain structure of collagen fibrils and chemical structure of the most common tripeptide sequence found in collagen, composed of Gly, Pro and Hyp sequences, (**b**) gelatin and its reaction with methacrylic anhydride to form photocrosslinkable gelatin–methacryloyl (GelMA).

Collagen (Figure 11a) is the most abundant protein in mammals, constituting up to one-third by weight of body proteins, as well as the main component of the ECM of many tissues, such as skin, bone, cartilage, tendons, blood vessels, and teeth, in which it contributes to the structural support, organization, and shape of tissues. Furthermore, it contains some adhesion motifs, such as RGD (Arg-Gly-Asp), which promote interaction with cells and regulate their proliferation, migration, and differentiation [237]. Around 28 types of collagen have been identified, and type I collagen is the most prevalent found in the ECM. This protein is arranged in three polypeptide chains, folded into a triple helix structure, with similar amino acid compositions. Glycine (Gly) is the most abundant amino acid present in the subunit chain (near 33%), forming with proline (Pro) and hydroxyproline (Hyp) the most common tripeptide sequence (10.5%) in collagen. Polypeptide chains wrap around each other with a right-handed rotation forming a very compact triple helix; specific hydrogen and covalent bonds between polypeptide chains stabilize the supramolecular structure. Collagen protein has a complex hierarchical conformation divided into four structures: primary (amino acid triplet), secondary (helix), tertiary (triple helix) and quaternary structure (fibrils) [238,239,240]. Type I mammalian collagen is currently used for many biomedical applications, thanks to its biocompatibility, biodegradability and suitable mechanical and cell-binding properties [240]. Moreover, the 3D architecture of crosslinked collagen-based hydrogels is suitable to mimic in vivo conditions of the tumor microenvironment [241].

**Figure 12:**
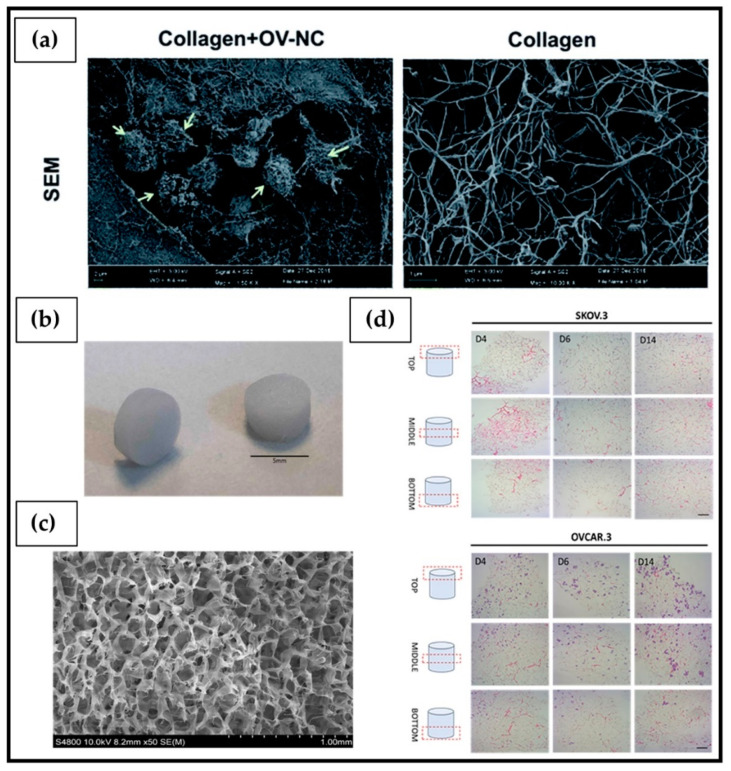
Collagen hydrogels. (**a**) SEM micrographs of collagen mesh (right) and OV-NC cells in collagen scaffolds (left) (yellow arrows indicate OV-NC cell clusters) [202]. (**b**) Jellyfish collagen-based sponges molded on 96-well plate (diameter of 5 mm), (**c**) SEM micrograph of scaffold porous structure, and (**d**) cell proliferation and migration throughout 3D collagen scaffold cross-section [203].

Collagen hydrogels designed by Ming et al. [202] were able to support OV-NC and OV-206 OC cell growth in vitro up to 16 days, with the formation of spheroidal cell aggregates (Figure 12a). Collagen enhanced the OC cell invasion, as well as cell ability to metastasize by upregulating the expression of MMPs and a5b1 integrin. Moreover, highly invasive OV-NC and OV-206 cells in collagen showed the overexpression of mesenchymal markers (i.e., N-cadherin, vimentin, and fibronectin) and transcriptional factors (Snail and Slug). OV-NC and OV-206 cells grown on the developed hydrogels showed higher resistance to carboplatin, 5-fluorouracil and paclitaxel, in comparison to the 2D cell culture reference. Scaffolds made of collagen deriving from alternative sources (e.g., marine), which allow one to avoid the high cost of production, immunogenicity, and chances of causing transmissible diseases, also offer a physiologically relevant tool for OC research and relevant preclinical drug testing. For instance, recent studies by Paradiso et al. [203] emphasized the ability of OVCAR3 and SKOV3 OC cells to invade and colonize marine collagen scaffolds throughout their whole cross-section (Figure 12b–d). Further studies were conducted to evaluate OC metastasis-related marker expression (e.g., mt1 mpp, col11a1, E-cadherin, vimentine, and yap) to understand if the 3D culture method promoted or repressed the metastatic properties of cancer cells. Gene expression of cellular markers was strongly influenced by the 3D microenvironment resulting in lower marker expression of most genes compared to a simple 2D culture system [242].

#### 6.2.2. Gelatin

The thermal denaturation of collagen triple helix leads to the formation of gelatin (Figure 11b). Commercial gelatin derives from various sources of collagen, such as hide, pig skin, bovine bones, and fish skin [243]. Depending on the preparation techniques, two distinct gelatins with different physical and chemical properties can be obtained. One is derived from the hydrolysis of collagen amide groups through an acid treatment, producing a limited density of carboxyl groups, with a resulting isoelectric point in the pH range 7.5–9.4 and a positive net charge (Type A gelatin). The other one is the product of an alkaline process displaying a high conversion of amide groups with an isoelectric point in the pH range 4.8–5.2 and a negative net charge (Type B gelatin) [155,244]. Gelatin-based hydrogels can be formed by either chemical or physical crosslinking. The easiest and fastest way to form hydrogels is by exploiting the heat-responsive properties of gelatin, which undergoes a reversible sol–gel transition by cooling the polymer solution below 35 °C [245]. Gelatin hydrogels with different physico-chemical and mechanical properties can be obtained by varying the type and concentration of a cross-linking agent [246]. Processing versatility, biocompatibility and bioactive properties make gelatin one of the most common polymers for the development of hydrogels tailored to several applications in drug delivery, tissue engineering, and medical textiles [247].

**Figure 13:**
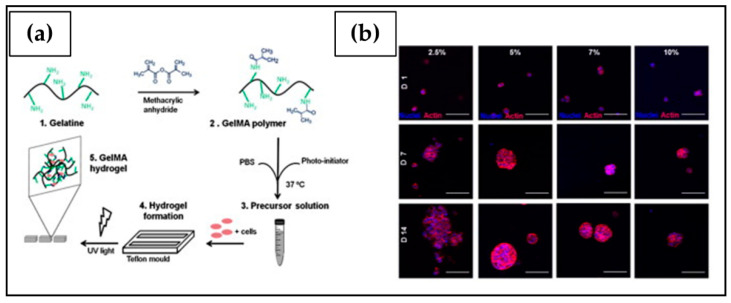
Gelatin–methacryloyl (GelMA) hydrogels. (**a**) GelMA-based hydrogel preparation: gelatin (1) and methacrylic anhydride reacted to form GelMA (2); GelMA is dissolved in PBS at 37 °C and mixed with the photoinitiator and cells (3); the cross-linking reaction is induced by UV light (4); the hydrogel is cut into smaller units (5). (**b**) Representative confocal laser scanning microscopy micrographs of cells embedded within GelMA hydrogels obtained from various polymer concentrations (*w*/*v*) and relevant stiffness (2.5%, 0.7 kPa; 5%, 3.4 kPa; 7%, 7.3 kPa and 10%, 16.5 kPa); nuclei are stained in blue and actin cytoskeleton in red (scale bars, 100 μm) [204].

Gelatin can be chemically modified through direct reaction with methacrylic anhydride (MA) in phosphate buffer solution (PBS) at 50 °C to obtain gelatin–methacryloyl (GelMA), which can be photocrosslinked to achieve high stability in physiological conditions [248]. According to studies carried out by Kaemmerer et al. [204], GelMA-based hydrogels crosslinked by UV irradiation in the presence of a photo-initiator (Irgacure) offer a low-cost, reproducible and tunable platform for 3D OC cell culture (Figure 13a). The developed scaffolds were able to support the formation of tumor spheroids with a defined size, resembling those present in ascites fluid. Polymer concentration (2.5–7% *w*/*v*) directly influenced hydrogel stiffness (0.5 ± 0.2 to 9.0 ± 1.8 kPa), and consequently cell spheroid morphology, metabolic activity, and proliferation. Indeed, a low GelMA concentration resulted in loose cell aggregates, whereas high GelMA concentrations led to smaller spheroids with a well-defined rounded shape. Spheroid proliferation from day 1 to 7 of cell culture was observed in all tested conditions (Figure 13b), and enhanced metabolic activity was detected in softer hydrogels. In addition, the incorporation of laminin-411 and hyaluronic acid (ECM components) into the scaffold matrix significantly improved spheroid formation and growth.

### 6.3. Synthetic Polymers

#### 6.3.1. RADA16-I

**Figure 14:**
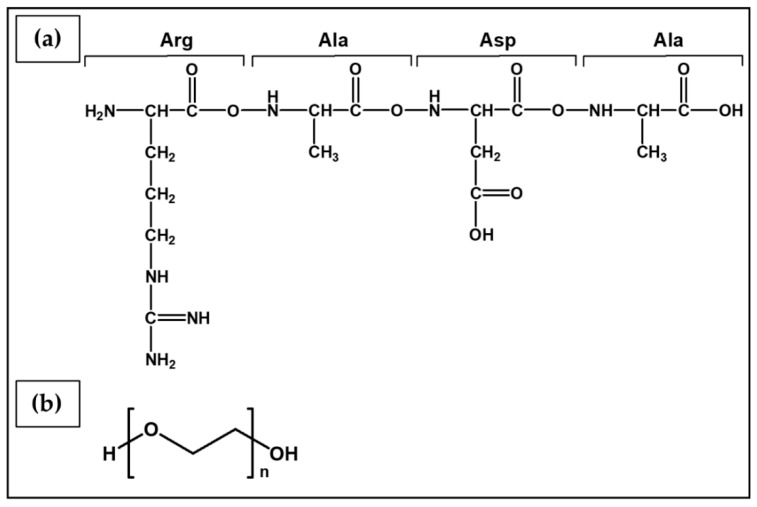
Chemical structure of synthetic polymers employed in hydrogel-based scaffolds for in vitro OC modeling. (**a**) Tetrapeptide sequence found in RIPA16-I peptide, and (**b**) poly(ethylene glycol) (PEG).

RADA16-I (Figure 14a) belongs to the ionic-complementary self-assembling peptides, a new class of synthetic biomaterials characterized by the alternation of amino acid residues with positive and negative charges separated by hydrophobic residues. Its primary structure consists of a tetrapeptide, arginine–alanine–aspartate–alanine (RADA), resembling the tripeptide RGD, an amino acid sequence that promotes cell adhesion. Peptide self-assembly in different electrolytic solvents results in a β-sheet secondary structure, thanks to both ionic and hydrophobic interactions [249]. The self-assembly process is strongly related to peptide sequence, concentration, pH, and the presence of salts, leading to the formation of different structures, such as fibers, membranes, and hydrogels. Based on charge distribution, it is possible to identify two different types of RADA peptide. In type I (RADA16-I: AcN-RADARADARADARADA-CNH_2_), one positively charged amino acid alternates with a negative one, whereas in type II (RADA16-II: AcN-RARADADARARADADA-CNH_2_) two positive residues alternate with two negatives. Upon exposure to aqueous solutions, the non-polar surfaces of two peptides face each other, originating a double-layered β-sheet nanofiber of about 10 nm in diameter that can further be elongated via an end-to-end fibril–fibril aggregation mechanism [250]. On the other hand, positive and negative charges are stuck together through intermolecular ionic interactions in a checkerboard-like manner [251]. The addition of monovalent ion salts or pH increase can enhance the process of hydrogel network formation, originating a nanofibril scaffold that can retain an extremely high content of water (>99% by weight) [251]. RADA peptides support the attachment and differentiation of various cellular types, such as neural cells, mesenchymal stem cells, chondrocytes, hepatocytes, cardiomyocytes, skin epithelial cells, endothelial cells, and OC cells [252]. They are also investigated for tissue regeneration to exploit their proven ability to repair spinal and neuronal tissue injuries [253].

**Figure 15:**
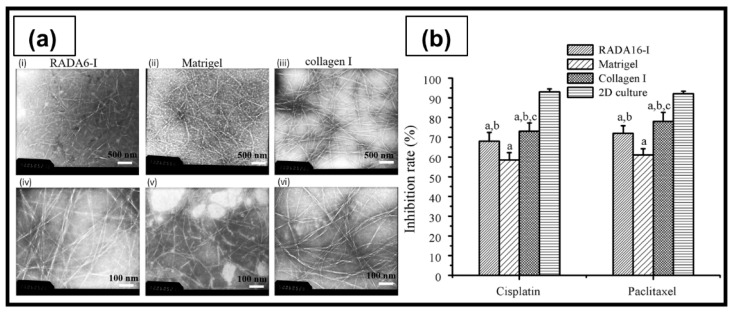
RADA16-I hydrogel. (**a**) Negatively stained TEM images showing the nanofiber morphology of RADA16-I (i,iv), Matrigel (ii,v), and collagen I (iii,vi) (scale bar, top panels (i–iii): ×8000; bottom panels (iv–vi): ×15,000). (**b**) Cisplatin and paclitaxel responses of chemosensitivity assay in HO-8910PM cells cultured in gel-cell clumps and common 2D flat cell plates: a (*p* < 0.01) compared with that of 2D cell culture model; b (*p* < 0.01) compared with that of Matrigel; c (*p* < 0.01) compared with that of RADA16-I hydrogel [206].

HO-8910PM OC cells cultured on RADA16-I nanofibrous scaffolds showed a spheroid or cluster morphology, with adhesion and migration properties comparable to what was observed in the case of type I collagen scaffolds [205]. The molecular expression of integrin β1, E-cadherin, and N-cadherin was quantitatively analyzed by immunohistochemistry and Western blotting assays. The obtained results indicate that HO-8910PM cells cultured in RADA16-I hydrogel formed proper cell–cell contact or intercellular junction, as well as more compact clusters than relevant 2D cell culture on tissue culture polystyrene. Moreover, RADA16-I nanofiber scaffold models were used to evaluate the cytotoxic activity of selected compounds (e.g., cisplatin, and paclitaxel) on a high metastatic human OC HO-8910PM cell line in comparison to the relevant counterparts of Matrigel, collagen I, and 2D culture [206]. The three kinds of investigated 3D OC models resulted in significantly higher IC50 values than those obtained through 2D flat culture, demonstrating increased chemoresistance (Figure 15).

#### 6.3.2. Poly(Ethylene Glycol) (PEG)

**Figure 16:**
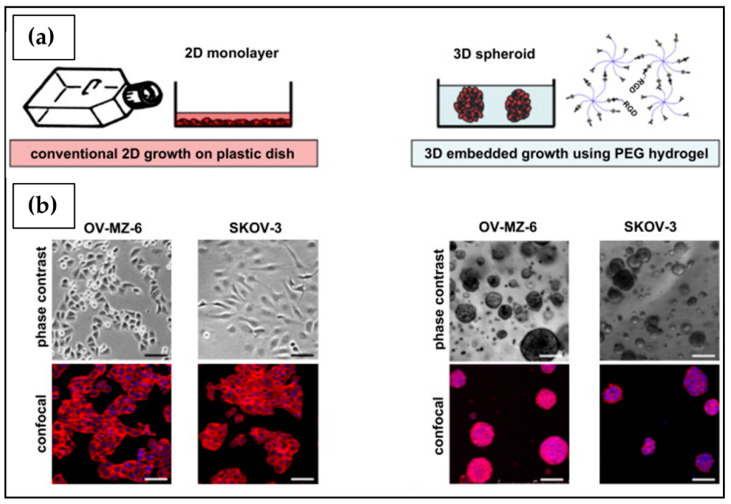
PEG-based hydrogel. (**a**) Schematic illustration of OC cells grown in a monolayer on traditional plastic surfaces (left) and in 3D as spheroids embedded within hydrogels (right). (**b**) OV-MZ-6 and SKOV-3 cells formed in 2D typical monolayers (left) and 3D systems (right), shown by phase contrast (top panel) and confocal (bottom panel; cell actin filaments stained with rhodamine phalloidin, nuclei with DAPI) microscopy images (scale bars, 75 μm) [208].

PEG (Figure 14b) is a hydrophilic polyether industrially produced through the polymerization of ethylene oxide [254,255]. Thanks to its well-documented properties, such as high solubility in aqueous media, biocompatibility, and the ability to effectively hide from the host’s immune system, the polymer has received approval from FDA for use in pharmaceutical, cosmetic, and biomedical applications [256,257]. Its derivatives, obtained through the functionalization of the terminal hydroxyl groups, are also widely used and studied for several applications [254,258]. In particular, PEG can be directly conjugated to another molecule, usually a drug or a protein. This covalent bond can mask the molecule to the host immune system (reducing immunogenicity and antigenicity), improve its solubility, decrease its renal clearance, and prolong its half-life [259]. Furthermore, PEG or its derivatives can be crosslinked to form hydrogels with tunable properties of great interest for in vitro 3D cancer modeling. Zhang et al. [207] evaluated the response of the HO8910 human OC cell line cultured in three kinds of hydrogels made of PEG with a different crosslinking degree, through reaction with poly(methyl vinyl ether-co-maleic acid); the hydrogel with higher rigidity showed better results in terms of tumor cell adhesion, migration, and invasiveness. Loessner et al. [208] fabricated a set of hydrogels obtained via factor XIII (FXIII)-catalyzed crosslinking between PEG and functionalized peptides. OV-MZ-6 and SKOV-3 OC cell lines embedded into these hydrogels organized in a spheroid form, similar to those found in ascites fluid (Figure 16). Matrix stiffness was varied as a function of polymer dry mass and influenced cell behavior: (i) proliferation of OV-MZ-6 cell spheroids within the hydrogels was decreased by increasing scaffold stiffness; (ii) the formation of irregular cell spheroids was observed only in softer hydrogels; (iii) quantitative cluster analysis showed smaller clusters in stiffer hydrogels, as well as a trend towards larger clusters in softer hydrogels.

## 7. Conclusions and Future Perspectives

The most commonly used in vitro cancer research model is still the 2D cell culture system. However, due to its limitations in recapitulating the cellular microenvironment present in vivo, 2D culture is increasingly being seen as an inefficient model. Three-dimensional cell culture systems represent an effective approach to new treatment strategies against OC, enabling tumor cells to assume their native morphology, as well as to establish cell–cell and cell–ECM interactions more faithfully representing those taking place in vivo. Thanks to their ability to absorb large amounts of water and mimic native ECM, hydrogel-based 3D scaffolds are attracting great interest in cancer research. Advanced crosslinking strategies and cutting-edge additive manufacturing approaches to process gelling polymers represent powerful tools for the development of scaffolds with predefined macroporous architecture. Natural and synthetic polymer hydrogel scaffolds have enabled significant progress in the study of OC progression and relevant drug screening, by developing 3D in vitro models that closely recapitulate pathophysiological features of native tissues.

Despite the great interest in the in vitro 3D modeling of OC witnessed by the fast-growing literature on this topic, critical aspects require further investigation to address unsolved problems. Particular attention should be paid in the near future to the development of relevant standards to enhance the experimental reproducibility of in vitro studies. Even if pioneering studies have recently shown the possibility of carrying out in vitro cancer cell culture for several weeks [97], most of the published articles in this area describe short-term experiments. The possibility of assessing the maintenance and evolution of tumor aggressiveness and metastatic potential over weeks/months as well as the possibility of testing in vitro dosage cycles of chemotherapy protocols are key aspects of future research in this context. In addition, a number of research articles on the in vitro modeling of other types of tumor have highlighted a significant influence of hydrogel stiffness on tumor behavior. However, this relationship has been poorly explored in the case of OC, and it surely represents a critical aspect to be investigated in the next future. Once the aforementioned issues and other relevant aspects of basic and applied research have been better defined, gold standards for in vitro tumor modeling will be available. This will allow for translating laboratory research findings to biomedical market and clinical practice.

In the near future, the use of 3D tumor models could become the standard for preclinical high-throughput screening of new chemotherapeutic agents and tumor pathophysiology investigation. Interdisciplinary research, continuous and constant technological and engineering advances, as well as the use of biomedical polymeric materials will certainly be at the forefront of the in vitro 3D tumor model revolution.

## Figures and Tables

**Table 1:** Peculiar features of 2D and 3D cell cultures for in vitro studies.

	2D Systems	3D Systems	Ref.
Morphology	Limited mimicking of the native tumor mass structure. Cells have a flat or stretched shape due to the attachment to rigid and flat substrates.	Cells grow in a 3D environment and maintain the typical tumor structure divided into three concentric zones of heterogeneous cell populations: an external proliferative zone, a central zone of quiescent cells, and an internal zone of necrotic cells.	[62]
Interaction	Limited cell–cell and cell–ECM interactions.	Physiological cell–cell contact similar to in vivo.	[63]
Perfusion	Unlimited cell access to oxygen, nutrients, metabolites and signaling molecules.	Gradients of oxygen, nutrients, metabolites, and signaling molecules.	[64]
Pharmacological action	More susceptible to drug action. Overexposure of cells to anticancer agents due to the absence of physical barriers.	Tumor morphology significantly affects the drug’s concentration throughout the tumor mass.	[65]
Gene/protein expression	Display different gene and protein expression levels compared to in vivo tissues.	Expression of tumor genes and proteins present in a relevant way even for long periods of culture.	[66]
Stiffness	Higher stiffness due to growth on a polystyrene tissue culture surface.	Lower stiffness more closely resembling that of native tissue.	[67]
Co-culture	Limited versatility.	High versatility.	[68]
Time of culture	Cells often proliferate at a faster rate than in vivo. Allow cells to grow up to 1 week.	Cells may proliferate at a different rate compared to 2D cell cultures. Allow cells to grow up for weeks.	[69]
Cost	Cheaper solution.	More expensive.	[5]
Availability	Commercially available tests and media.	Limited number of commercially available tests.	[70]
Maintenance and handling	Easier maintenance and manipulation.	Time consuming. Greater difficulty in carrying out methodological techniques.	[5,71]

**Table 2:** Advantages and disadvantages of scaffold-free cell culture techniques.

	**Ultra-Low Attachment** 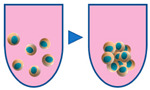	**Hanging Drop** 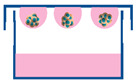	**Agitation-Based** 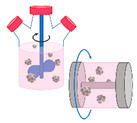
Method	Cells are cultured on a substrate having non-adhesive properties (e.g., hydrophilicity, uncharged and concave surface). Cell–cell interactions are easier to be established than cell-surface ones.	Cells aggregate spontaneously at the apex of a droplet of culture medium, suspended on the lid of a multi-well plate.	Cell aggregates are maintained in suspension in culture medium and their adhesion to the bioreactor surface is prevented by a continuous agitation system (e.g., mechanical stirrer or rotating-wall).
Advantages	Low-cost. High throughput screening. It is possible to control size uniformity with specialized equipment.	Low-cost. May not need specialized equipment. Good shape and size control.	Long term culture. Large-scale production. High control of culture conditions.
Disadvantages	Long term culture is complex. Plate-coating procedure may be laborious.	No long term culture. Not stable system. Difficulty in medium replacement and compounds addition. Dehydration risk. No large spheroids. Labor intensive.	Require specialized equipment. Shear stresses acting on cells. Poor control of spheroid shape and size. No individual compartment for each spheroid. Difficulty to collect cells.
Reference	[75,76,77,78,79,80]	[81,82,83,84]	[86]

**Table 4:** Three-dimensional culture systems of OC cells combined with polymeric materials.

Polymer(s)	Hydrogel Formation	Cell Line(s)	Outcomes	Ref.
Chitosan/Alginate	Chemical crosslinking: N-succinyl chitosan mixed with oxidized alginate at room temperature	SKOV3	High reproducibility of hydrogel geometry. OC cells exhibit enriched expression of tumor-associated antigens.	[197]
Chitosan/Bacterial Cellulose	Physical crosslinking: single-step mixing between cellulose and chitosan	A2780	OC cells adhered to the surface and infiltrated deep into the scaffold with a strong cell-scaffold interaction, confirmed by the decrease in the mRNA level of Notch receptor.	[198]
Agarose	Thermal crosslinking at 90 °C	SKOV3	Increased growth and malignancy of the tumor mass in comparison to 2D culture, demonstrated by the upregulated expression of hypoxic and pro-angiogenic factors.	[199]
Alginate/Marine Collagen/Agarose	Physical crosslinking: sequential mixing of sodium alginate solution, marine collagen solution, and agarose solution	A2780	The 3D model allowed a long-time culture, with higher cell proliferation compared to 2D systems, and promotion of gene expressions of ICAM-1, IL-7, TARC and GM-CSF.	[200]
Alginate	Physical crosslinking: alginate solution is dripped into a CaCl_2_ gelling bath to form alginate beads	SKOV3	Cells embedded in alginate beads and cultured in a fluid-dynamic bioreactor (MIVO^®^) exhibited responses to cisplatin action that closely resembled those obtained in the xenograft model.	[87]
Alginate/Poly(ehtylene glycol) (PEG)/Poly(methyl vinyl ether-alt-maleic acid) (PMVE-*alt*-MA)	Chemical/physical crosslinking: double-network hydrogels, consisting of PEG covalently crosslinked PMVE-*alt*-MA and alginate ionically cross-linked with Sr^2+^, Ca^2+^, or Fe^3+^	SKOV3	Variation of cation led to differences in scaffold pore size, mechanical and swelling properties. Fe^3+^ ionically crosslinked alginate hydrogels had higher porosity and swelling degree that significantly improved cell malignancy and tumorigenicity.	[201]
Mammalian Collagen	Thermal crosslinking at 37 °C for 2 h	OV-NC OV-206	Formation of spheroids with high cell viability and low growth rate. Improved cell invasion/motility by upregulating the expression of MMP, integrin a5b1 and mesenchymal markers (N-cadherin, vimentin and fibronectin) and transcription factors (Snail and Slug). In addition, 3D cultures revealed significantly improved drug resistance to chemotherapy.	[202]
Marine Collagen	Chemical crosslinking: lyophilized collagen crosslinked using 1-ethyl-(3-3- dimethylaminopropyl) carbodiimide hydrochloride	SKOV3 OVCAR3	The hydrogel interconnected pores network allowed colonization of both cell lines, which showed altered expression of some bio markers in a 3D environment compared to 2D culture.	[203]
Gelatin methacryloyl (GelMA)	Photo crosslinking: GelMA-based hydrogels crosslinked by UV irradiation in the presence of a water-soluble photo-initiator (Irgacure)	OV-MZ-6	Cell proliferation affected by the stiffness of the support; incorporation of the laminin-411 and hyaluronic acid into the hydrogel further stimulated spheroidal growth.	[204]
RADA16-I	Peptide self-assembling in ultrapure water	A2780 A2780/DDP SKOV3	The peptide nanofibers exhibited some biological characteristics similar to type I collagen, and allowed the maintenance of the tumorigenic cell phenotype and higher cell resistance to 5-FU, paclitaxel, and curcumin, compared with 2D culture.	[205]
RADA16-I	Peptide self-assembling in ultrapure water	HO8910PM	Cells cultured in RADA16-I hydrogel organized as spherical agglomerates with well-organized and regularly arranged nuclei. Formation of compact cell–cell or cell–ECM interactions similar to 3D cell culture in Matrigel.	[206]
PEG	Chemical crosslinking with poly(methyl vinyl ether-co-maleic acid)	HO8910PM	Adhesion, proliferation and migration of tumor cells closely related to hydrogel stiffness, which could be adjusted by changing the crosslinking degree.	[207]
PEG	Chemical crosslinking reaction by thrombin-activated factor XIII substrates	OV-MZ-6 SKOV3	3D matrices allowed long-term cultures, cell–ECM interactions implicated in cancer development, and were suitable for anticancer drug screening.	[208]
Peptide amphiphiles/Protein (keratin or fibronectin)	Peptide-protein self-assembling	OVCAR4	Self-assembled hydrogels supported the formation of tumor spheroids surrounded by an F-actin network, which promoted cell–cell interactions.	[209]

## Data Availability

Not applicable.
